# Characteristics of Polyphenols of Black Hulless Barley Bran and Its Anti-Diabetic Activity

**DOI:** 10.3390/foods14172994

**Published:** 2025-08-27

**Authors:** Junlin Deng, Tinghui Liu, Chen Xia, Litao Tong, Chunmei Gu, Zhiqiang Shi, Yuehang Yang, Ruiling Zhan, Zhuoya Xiang, Jian Chen, Yan Wan, Manyou Yu

**Affiliations:** 1 Institute of Agro-Products Processing Science and Technology (Institute of Food Nutrition and Health), Sichuan Academy of Agricultural Sciences, Chengdu 610066, China; junlinr@scsaas.cn (J.D.); gnspxiachen@scsaas.cn (C.X.); shizhiqiang@163.com (Z.S.); xiangzy@scsaas.cn (Z.X.); jianchen11@scsaas.cn (J.C.); 2 Ganzi Academy of Agricultural Sciences, Kangding 626000, China; liutinghui@yeah.net (T.L.);; 3 Institute of Food Science and Technology, Chinese Academy of Agricultural Sciences/Key Laboratory of Agro-Products Processing, Ministry of Agriculture, Beijing 100193, China; tonglitao@caas.cn; 4 School of Pharmacy and Bioengineering, Chengdu University, Chengdu 610106, China; yyhwsyy@163.com

**Keywords:** black hulless barley bran, polyphenols, T2DM, intestinal microbiota, transcriptome

## Abstract

Polyphenols play a crucial role in promoting human health. This study aims to investigate the polyphenols of black hulless barley bran (HBP) and evaluate their anti-diabetic mechanisms in vivo. Using UPLC-QTOF-MS/MS, 27 compounds were identified in HBP, including four phenolic acids, 14 flavonoids, and nine anthocyanidins. High contents of Chrysoeriol 7-O-glucuronide (42.09 mg/g), Cyanidin 3-O-glucoside (21.02 mg/g), and Cyanidin 3-O-(6″-O-malonyl)-glucoside (24.45 mg/g) were quantified via UPLC in HBP. Administration of HBP significantly reduced fasting blood glucose (FBG), improved glucose intolerance and lipid profiles, and alleviated liver and pancreatic damage in type 2 diabetic (T2DM) mice. Furthermore, it enhanced serum antioxidant enzyme activities and modulated inflammatory cytokines. Transcriptomic analysis revealed that HBP influenced signal transduction and the immune system, particularly in key signaling pathways, including Hippo, TGF-beta, HIF-1, and p53, associated with T2DM. Although HBP had minimal impact on gut microbiota diversity and SCFA levels, it presents a promising candidate for T2DM intervention through its multifaceted mechanisms.

## 1. Introduction

Type II diabetes mellitus (T2DM) is a chronic metabolic disease characterized by metabolic disorder, including hyperglycemia, hyperlipidemia, and insulin resistance. Recent data from the International Diabetes Federation (IDF Diabetes Atlas 11th edition 2025) indicate that 589 million adults (20–79 years) are living with diabetes worldwide, and this number is predicted to rise to 853 million by 2050. Diabetes caused at least USD 1 trillion dollars in health expenditure—a 338% increase over the last 17 years. The largest number of individuals affected by T2DM accounts for 96% of all diabetes cases [1]. Diabetes raises serious concerns, including life-threatening, disabling, and costly complications, as well as a reduction in life expectancy [2,3]. Given the increasing prevalence and severe health problems associated with diabetes, the use of herbs and botanicals as dietary interventions is urgently needed to control glycemic levels and manage diabetic complications [4]. Numerous bioactive compounds found in plants, including polysaccharides, polyphenols, flavonoids, triterpenoids, and their glycosides, have garnered attention due to their excellent anti-diabetic effects and long-term safety [5,6].

Compared with Chinese Han participants, Tibetan Chinese had significantly lower crude prevalence of diabetes (Tibetan: 4.3% [95% CI, 3.5–5.0%]; Han: 14.7% [95% CI, 14.6–14.9%]) [7]. This phenomenon might be associated with their lifestyles and dietary patterns, such as higher levels of physical activity, consuming hulless barley and drinking Tibetan tea. Hulless barley (*Hordeum vulgare* L. *var. Nudum* Hook.f.) is an ancient and unique cereal crop that serves as a stable food source and animal feed in the Qinghai–Tibet Plateau of China. It possesses a high content of protein, vitamins, and dietary fiber, while having a low content of sugar and fat, which aligns with the requirements of a modern and healthy lifestyle [8]. Furthermore, the high levels of β-glucan, along with abundant flavonoids, phenolic acids, and anthocyanins, endow hulless barley with anticancer properties, antioxidant capabilities, and a reduction in the risk of chronic diseases [9,10,11]. However, Šimić, et al. [12] and Xiang, et al. [13] reported that the phytochemical components, including β-glucans, dietary fiber, flavonoids, and anthocyanin, were detected in hulless barley bran, which is often discarded during the grain production process.

In our previous study, the ethanol extracts of black hulless barley bran, which contain an abundance of phenolic compounds, exhibited an inhibitory effect on α-glucosidase [14]. The ethyl acetate extracts of hulless barley bran, which contain a large number of phenolic compounds, demonstrated a high inhibition rate against both α-glucosidase and α-amylase [15]. Additionally, Gujral, et al. [16] noted that hulless barley bran could ameliorate diabetic symptoms in vivo, possibly due to its high dietary fiber content. Therefore, existing evidence suggests that components in hulless barley bran tend to regulate blood glucose levels; however, the potential anti-diabetic mechanisms of hulless barley bran remain to be elucidated [17]. In the present study, black hulless barley bran is selected to elucidate the characteristics of polyphenolic extracts and to investigate their hypoglycemic effects in a T2DM mouse model. This research aims to provide deeper insights into the components of hulless barley bran and their potential health benefits for T2DM, along with the possible underlying mechanisms.

## 2. Materials and Methods

The Longzi variety of hulless barley, characterized by its black grain color, was provided by Tibet Chunguang Food Co., Ltd. (Lhasa, China). The black hulless barley bran was ground into powder and passed through a 60-mesh sieve, then stored at −20 °C for further testing.

### 2.1. Chemicals and Reagents

Streptozotocin (STZ) was purchased from American Sigma Company (St. Louis, MO, USA). The ELISA kit was obtained from Shanghai Yuanye Biotechnology Co., Ltd. (Shanghai, China). Additionally, formic acid and ethanol were sourced from Chengdu Kelong Chemical Reagent Factory (Chengdu, China). All chemicals used were of analytical grade or higher.

### 2.2. Preparation of Polyphenols of Black Hulless Barley Bran (HBP)

A total of 500 g of bran powder was extracted using 3 L of 80% ethanol containing 1% formic acid for 40 min at 40 °C, with the assistance of ultrasound using the KH2200DE ultrasonic instrument (Kunshan Ultrasound Instrument Co., Kunshan, China). The mixture was then filtered, and the residue was subjected to two additional extractions. The three filtrates were combined and evaporated to a final volume of 500 mL at 50 °C under vacuum using a rotary evaporator (Hei-VAP; Heidolph Instruments, Schwabach, Germany). The ethanol extracts of black hulless barley bran were stored at −20 °C for subsequent purification.

AB-8 macroporous resin was purchased from Chengdu Kelong Chemical Reagent Factory (Chengdu, China). The resin was washed with three bed volumes (BV) of distilled water until it became colorless. Subsequently, it was soaked in three BV of ethanol for 24 h and then repeatedly rinsed with distilled water until no ethanol odor was detected. Following this, the resin was soaked in five BV of 4% HCl for 2 h and then washed with distilled water until it reached near neutrality. Finally, the resin was soaked in five BV of 4% NaOH for 2 h and rinsed until it was near neutral.

The wet AB-8 resin was packed into a glass chromatography column (diameter 5 cm × height 43 cm), rinsed repeatedly with distilled water, and soaked in water for 20 min. The ethanol extracts of black hulless barley bran were then introduced into the column at a flow rate of 1 BV/h and allowed to adsorb for 1 h. Subsequently, the column was washed with 4 BV of 0.1% hydrochloric acid at a flow rate of 2 BV/h and the eluent was discarded. Following this, the column was washed with 5 BV of 80% ethanol. The eluate was collected, concentrated under reduced pressure at 50 °C, and freeze-dried at −80 °C to obtain the polyphenols of black hulless barley bran (HBP), which were stored in a sealed container at −20 °C for later analysis.

### 2.3. Phytochemical Composition Analyses of HBP

The qualification of HBP was performed using an UPLC system (Waters Corporation, Milford, MA, USA) equipped with PDA detector, coupled with a Waters Xevo G2-XS QTOF-MS/MS spectrometer (Waters, Manchester, UK) and fitted with an ESI source operating in negative mode. The Waters BEH C_18_ column (2.1 mm × 100 mm, 1.7 μm particle size) was employed for chromatographic separation. The UPLC operation conditions were set as follows: 30 °C of column temperature, 1 μL of volume, and 0.3 mL/min of flow rate, 320 nm and 350 nm of PDA spectra. Solvents A (water + 0.1% formic acid) and B (acetonitrile) were utilized under the following gradient conditions: 0–3 min (5–8%), 3–8 min (8–15%), 8–15 min (15–50%), 15–17 min (50–70%), and 17–19 min (70–95%). The MS parameters were configured as follows: capillary voltage of 2.5 kV, desolvation gas flow of 600 L/h, cone gas flow of 50 L/h, desolvation temperature at 250 °C, cone voltage of 40 V, source default voltage of 80 V, and mass scanning range from *m*/*z* 50 to 1500.

The analysis of characteristic phenolic compounds was determined using an Agilent 1290 series UPLC system (Agilent Technologies, Santa Clara, CA, USA) equipped with a diode array detector (DAD). Separation was achieved on a Poroshell 120 PEP column (4.6 mm × 2.1 mm, 1.7 μm: Agilent Technologies, Santa Clara, CA, USA). The detection was carried out at a column temperature of 30 °C with a 5 μL injection volume at a flow rate of 0.8 mL/min. The detection wavelengths were set at 254, 280, 320, 350 and 525 nm. A mobile phase consisting of 0.1% formic acid in water (A) and acetonitrile (B) was utilized, with the following gradient program: 0–10 min for 5–10%, 10–20 min for 10–20%, 20–35 min for 20–40%, 35–40 min for 40–70%, and 40–45 min for 70–95% B. The eight polyphenols were quantified against their corresponding standard compounds and expressed as polyphenols equivalents per gram of dry weight (µg/g DW).

The total anthocyanin content (TAC) was analyzed using the high-performance liquid chromatography (HPLC) method. The total phenolic content (TPC) and total flavonoid content (TFC) were assessed using the Folin–Ciocalteu and aluminum chloride (AlCl_3_) colorimetric method, respectively. Detailed descriptions of these procedures can be found in our previously published article [14].

### 2.4. Animals and Experiments

#### 2.4.1. Animal

A total of 48 male SPF C57BL/6 mice, each weighing approximately 15 ± 2 g and aged 6 weeks, were procured from Chengdu Dashuo Experimental Animal Co., Ltd. (Chengdu, China) and Beijing Vital River Laboratory Animal Technology Co., Ltd. (Chengdu, China). The mice were acclimatized to their new environment for 3 days, during which they had free access to food and water, with three mice housed per cage in an environmentally controlled room maintained at 24 ± 2 °C and 40% to 60% of relative humidity, following a 12-h light/dark cycle. Subsequently, the mice were randomly assigned to either a normal control group (NC, *n* = 12) receiving a basic diet (see Supplementary Table S1) or a diabetic group (*n* = 36) receiving a high-fat diet (see Supplementary Table S1), and they were fed for a duration of 40 days. All animal procedures and laboratory conditions in this study adhered to the guidelines set forth by the Animal Care and Use Committee (Approval No. 2024LS056) at Sichuan Normal University, China. After 5 weeks, the mice were deprived of food and water for 12 h and were injected intraperitoneally once with STZ (60 mg/kg body weight). Simultaneously, the normal mice received an equivalent volume of citrate buffer (0.1 mol/L, pH 4.5). Three days after STZ administration, fasting blood glucose (FBG) levels were measured through tail-vein nick. The diabetic model was considered successful when FBG level surpassed 11.1 mmol/L [18]. Then the diabetic model mice were randomly divided into three groups: the diabetic control group (CM, *n* = 12), and two dosage groups, namely the diabetic high-dose HBP group (HDP, 400 mg/kg BW HBP, *n* = 12) and the diabetic low-dose HBP group (LDP, 100 mg/kg BW HBP, *n* = 12). Meanwhile, NC and CM mice received an equivalent volume of 0.9% physiological saline by gavage. The experimental period lasted for 42 days. Feces were collected 48 h prior to sacrifice and stored at −80 °C. In the final stage of treatment, the mice were fasted for 12 h prior to sacrifice. Blood samples were collected and centrifuged at 3500 rpm for 15 min to obtain serum for subsequent analyses. Additionally, the tissues of livers and pancreases were immediately excised and stored at −80 °C.

#### 2.4.2. Measurement of Fasting Blood Glucose (FBG), and Oral Glucose Tolerance Test (OGTT)

Fasting blood glucose (FBG) was measured using an Accu-Chek Performa blood glucose meter (Roche Diagnostics, Mannheim, Germany). Measurements were taken with the blood glucose meter from the tail vein after an 8-h fasting period without water, conducted weekly from weeks 1 to 5. The oral glucose tolerance test (OGTT) was performed one week prior to euthanasia. Following a 12-h fast, the mice were gavaged with a glucose solution at a dosage of 2 g/kg. Tail-vein blood samples were then collected, and blood glucose levels were assessed at 0, 15, 30, 60, and 120 min post-glucose administration. The area under the curve (AUC) during the OGTT was subsequently calculated [19].

#### 2.4.3. FINS, HPMA-IS, HPMA-IR

The mice were subjected to fasting overnight and were not allowed to drink water for 12 h before measuring their fasting blood glucose (FBG). Subsequently, the eyeballs were removed and blood samples were collected. The blood was allowed to stand for 30 min and then centrifuged at 4 °C at 5000 rpm for 15 min. The supernatant was transferred to a sterile centrifuge tube and immediately stored in a −80 °C freezer for insulin level measurement. Fasting insulin levels (FISN) were quantified using ELISA kits according to the instructions provided with the Mouse INS ELISA KIT. The homeostasis model assessment of insulin resistance index (HOMA-IR) and the homeostasis model assessment of insulin sensitivity index (HOMA-IS) were evaluated using the following formulas [20]:

HOMA-IR = FBG (mmol/L) × FISN (μU/mL)/22.5

HOMA-IS = 1/[FBG (mmol/L) × FISN (μU/mL)]

#### 2.4.4. Organ Index of Liver, Pancreas and Epididymal Fat

The liver, pancreas, and epididymal fat weights were detected to calculate the organs index, expressed as organ mass (g) to body mass (g) ratio.

#### 2.4.5. Lipemia, Antioxidant and Anti-Inflammatory Assay

The serum samples were analyzed using a fully automated biochemical analyzer to quantify the levels of total cholesterol (TC), triglycerides (TG), high-density lipoprotein cholesterol (HDL-C), low-density lipoprotein cholesterol (LDL-C), aspartate aminotransferase (AST), and alanine aminotransferase (ALT). Liver tissues samples were homogenized with physiological saline (1:9, *w*/*v*) and then centrifuged at 3000 rpm for 10 min at 4 °C. The supernatants were collected to assess antioxidant assays. The antioxidant indicators in liver and serum, namely superoxide dismutase (SOD), malondialdehyde (MDA), and catalase (CAT), were detected via enzyme-linked immunosorbent assay kits. These kits were also employed to measure serum inflammatory factors, including tumor necrosis factor alpha (TNF-α), interleukin 6 (IL-6), and the anti-inflammatory factor interleukin 6 (IL-4).

#### 2.4.6. Histopathological Analysis

The middle lobe of the liver was fixed with 40% paraformaldehyde, dehydrated and embedded in paraffin wax [20]. It was then sectioned into 4-µm thick slices (HM325, Thermo Fisher Scientific, Waltham, MA, USA) and mounted on glass slides. The sections underwent a gradient dewaxing process followed by hematoxylin–eosin (HE) staining and were subsequently sealed with neutral resin for the observation of histomorphological changes under a microscope (Primo Star, ZEISS, Oberkochen, Germany). Cell measurements were conducted on cells using panoramic slice scanner (PANNORAMIC SCAN, 3DHISTECH, Budapest, Hungary) and slide viewer (Slide Viewer 2.5.0, 3DHISTECH, Budapest, Hungary).

#### 2.4.7. Determination of SCFAs

A 20-mg sample of fecal matter, added with 1 mL phosphoric acid (0.5% *v*/*v*), was milled 1 min at 30 Hz using MM400 ball crusher (Retsch, Haan, Germany), vortexed for 10 min and ultrasonicated for 5 min at 4 °C, then centrifuged at 12,000 r/min for 10 min at 4 °C. A volume of 100 μL of the supernatant was mixed with 500 μL of MTBE (containing an internal standard) solution vortexed for 3 min, subsequently ultrasonicated for 5 min at 4 °C, and then centrifuged again at 12,000 r/min for 10 min at 4 °C. The supernatant was collected and filtered using a 0.22-μm organic membrane filter for GC-MS/MS analysis [21]. The analysis of short-chain fatty acids (SCFAs) was employed by using an Agilent 7890B gas chromatograph coupled with a 7000 D mass spectrometer, equipped with a DB-FFAP column (30 m × 0.25 mm × 0.25 µm, J & W Scientific, Newark, DE, USA). The operation conditions were set as follows: helium at a flow rate of 1.2 mL/min, front inlet mode at a split ratio of 5:1, 1 µL of injection volume, 250 °C of front injection temperature, 250 °C of ion source temperature. The oven temperature was held at 50 °C for 1 min, then raised to 220 °C at a rate of 18 °C/min and kept for 5 min.

#### 2.4.8. High-Throughput 16S rDNA Sequencing of Gut Microbiota

The concentration and purity of total genomic DNA extracted from fecal samples using the CTAB/SDS method were evaluated by agarose gel electrophoresis. The V3–V4 regions of the 16S rRNA gene were amplified using the following primers: (5′-TCGTCGCAGCGTCAGATGTGTATAAGAGAGAGCCTACGGGNGGCWGCAG-3′ and 5′-GTCTCGTGGCTCGGAGATGTGTAAGAGAGAGACGACTACHVGG TATCTAATCC-3′). Qualifying PCR products detected via electrophoresis on a 2% agarose gel were purified using magnetic beads and quantified with an enzyme label. The target DNA fragment was recovered using a gel recovery kit (Qiagen, Hilden, Germany). The TruSeq^®^ DNA PCR-Free Sample Preparation Kit was employed for library construction, which was quantified using Qubit and Q-PCR. After passing quality control, the library was sequenced on the Illumina NovaSeq 6000 platform.

The sample-specific data were separated from the offline data according to the barcode sequence and the PCR amplification primer sequence. The original reads were filtered using Fastp (v0.22.0, https://github.com/OpenGene/fastp, accessed on 14 November 2024) to obtain high-quality reads. High-quality dual-end reads were spliced using FLASH (v1.2.11, http://ccb.jhu.edu/software/FLASH/, accessed on 14 November 2024) to generate high-quality tag data (Clean Tags). Chimeric sequences were detected and removed from the tag sequences by comparing them with the species annotation database through VSEARCH (v2.22.1, https://github.com/torognes/vsearch/, accessed on 14 November 2024) to obtain the final effective tags. The sequence data were then filtered, trimmed, and further classified into operational taxonomic units (OTUs) based on the principle of 97% sequence similarity. Species annotation was completed using the Mothur (v1.48) method and the SSUrRNA database (SILVA138.1, http://www.arb-silva.de/, accessed on 14 November 2024) to obtain taxonomic information and calculate the community composition of each sample at various taxonomic levels. Subsequent alpha diversity and beta diversity analyses were conducted based on the homogenized data obtained from processing each sample’s data with the smallest amount of data as the standard.

#### 2.4.9. Transcriptome Sequencing of the Hepatic

Total RNA was extracted from the tissue using Trizol reagent (Invitrogen Life Technologies, Carlsbad, CA, USA). The quality of the RNA was assessed using a 5300 Bioanalyzer (Agilent Technologies, Santa Clara, CA, USA) and quantified with the NanoDrop Technologies(ND-2000, Thermo Scientific, Waltham, MA, USA). RNA purification, reverse transcription, library construction and sequencing were conducted at Shanghai Majorbio Bio-pharm Biotechnology Co., Ltd. (Shanghai, China) according to the manufacturer’s instructions. The RNA-seq transcriptome library was prepared using the Illumina Stranded mRNA Prep, Ligation (San Diego, CA, USA) protocol with 1 μg of total RNA. Following library preparation, sequencing was performed on DNBSEQ-T7 platform (PE150) using DNBSEQ-T7RS Reagent Kit (FCL PE150) version 3.0. (T7 sequencing platform). The resulting data underwent quality control, read mapping, and expression analysis prior to further differential analysis. Data analysis was conducted on the Majorbio Cloud Platform (www.majorbio.com (accessed on 14 November 2024)).

#### 2.4.10. Quantitative Real-Time PCR (RT-qPCR) Validation

Total RNA was extracted from liver tissue using Trizol reagent, followed by the generation of cDNA through a reverse transcription process. The cDNA samples were analyzed using real-time fluorescence PCR (ABI7300, Thermo Scientific, Waltham, MA, USA). The relative mRNA expression of the target genes was assessed using the 2^−ΔΔCt^ method. The specific primers utilized are listed in Supplementary Table S2.

### 2.5. Statistical Analysis

The results are presented as mean ± standard deviation (SD). A one-way analysis of variance (ANOVA) with Dunnett’s *t*-test was conducted to assess statistical differences, with *p* < 0.05 with statistically significant. All statistical analyses were carried out using GraphPad Prism version 9.5 (GraphPad Prism, San Diego, CA, USA) and R version 4.2.0.

## 3. Results

### 3.1. Analysis of the HBP Composition

The UPLC-QTOF-MS/MS analysis characterized and identified 27 compounds in HBP, including one phenolic acid, 17 flavonoids (three catechins, eight flavonols and their glucosides, six flavones and their glucosides), and nine anthocyanidins and their derivatives, using authentic standards, published references, and MS/MS fragmentation patterns (Table 1). The MS spectra of the identified compounds in HBP are provided in Supplementary Figure S1. According to Table 1, the four phenolic acids fall under the category of catechins. The predominant flavonoids in the extracts are derivatives of chrysoeriol and luteolin, with the sugars being glucoside and glucuronide. All nine anthocyanidins are categorized as cyanidin derivatives, and the sugars present include glucoside, galactoside and glucuronide. Furthermore, the TAC, TPC, and TFC in HBP were quantified at 55.39, 242.336, and 138.8 mg/g, respectively (Table 2). Additionally, eight individual phenolic compounds were quantified in HBP, including protocatechuic acid, luteolin 7-O-glucuronide, luteolin-7-O-glucoside, chrysoeriol 7-O-glucuronide, luteolin, chrysoeriol, cyanidin 3-O-glucoside, and cyanidin 3-O-(-O-malonyl)-glucoside, as identified through HPLC-Q-TOF-MS/MS analysis. Chrysoeriol 7-O-glucuronide (42.09 mg/g), cyanidin 3-O-glucoside (21.02 mg/g), and cyanidin 3-O-(6′-O-malonyl)-glucoside (24.45 mg/g) exhibited higher concentrations in HBP compared to other individual phenolic compounds.

**Table 1:** Qualification of components by UPLC-Q-TOF-MS/MS of HBP.

Peak No.	Rt (min)	[M − H]^−^ (*m*/*z*)	[M + H]^+^ (*m*/*z*)	Fragment Ions	Formular	Identified Compounds
1	2.783	153.0177		109	C_7_H_6_O_4_	Protocatechuic acid
2	2.884	593.1298		425,407,289,243,177,000	C_30_H_26_O_13_	(Epi)gallocatechin-(epi)catechin
3	5.176	577.1251		407,289,125	C_30_H_26_O_12_	(Epi)catechin-(epi)catechin
4	5.76	289.0689		245,109	C_15_H_14_O_6_	Catechin
5	9.571	447.0848		357,327,297,285	C_21_H_20_O_11_	Orientin or Isoorientin
6	10.688	609.1411		301,300,271,255,243	C_27_H_30_O_16_	Quercetin 3-O-rutinoside
7	10.733	431.0968		341,311,283	C_21_H_20_O_10_	Isovitexin or vitexin
8	10.916	463.0864		301,300,271,255	C_21_H_20_O_12_	Quercetin-3-O-glucoside
9	11.02	461.0702		285,133	C_21_H_18_O_12_	Luteolin 7-O-glucuronide
10	11.06	447.0892		285,284	C_21_H_20_O_11_	Luteolin-7-O-glucoside
11	11.659	607.1614		299,284,255,227	C_27_H_28_O_16_	Chrysoeriol 7-O-rutinoside
12	11.984	475.088		299,284,256	C_22_H_20_O_12_	Chrysoeriol 7-O-glucuronide
13	12.052	461.1054		299,255	C_21_H_18_O_12_	Chrysoeriol 7-O-glucoside
14	12.956	489.1028		285	C_24_H_26_O_11_	Luteolin 7-O-(6″-O-acetyl)-glucoside
15	13.134	285.0407		133	C_15_H_10_O_6_	Luteolin
16	13.182	489.0982		474,298,283,255	C_24_H_26_O_11_	Chrysoeriol 7-O-methylglucuronide
17	13.757	503.1145		488,312,283,255	C_24_H_24_O_12_	Chrysoeriol 7-O-(6″-O-acetyl)-glucoside
18	14.21	299.0557		284,256,227	C_16_H_12_O_6_	Chrysoeriol
19	5.954		611.25156	287	C_27_H_31_O_16_	Cyanidin 3-O-diglucoside
20	6.409		449.1387	287	C_21_H_21_O_11_	Cyanidin 3-O-glucoside
21	7.375		433.1442	271	C_21_H_21_O_10_	Pelargonidin 3-O-glucoside
22	8.152		535.1491	287	C_24_H_23_O_14_	Cyanidin 3-O-(6″-O-malonyl)-galactoside
23	9.173		535.1491	287	C_24_H_23_O_14_	Cyanidin 3-O-(6″-O-malonyl)-glucoside
24	10.432		621.1627	287	C_27_H_25_O_17_	Cyanidin3-O-(3″,6″-O-dimalonyl)-glucoside
25	10.885		563.1796	287	C_25_H_23_O_15_	Cyanidin 3-O-(6″-O-succinyl)-glucuronide
26	11.287		563.1893	287	C_25_H_23_O_15_	Cyanidin 3-O-(3″-O-succinyl)-glucuronide
27	11.687		649.2008	287	C_29_H_29_O_17_	Cyanidin 3-O-(3″,6″-O-disuccinyl)-glucoside

**Table 2:** Quantification of HBP.

Compounds	Equation	*R* ^2^	Linear Range (µg/mL)	Content (Mean ± SD, mg/g)
protocatechuic acid	*y* = 19.293*x* − 34.878	0.9997	1.95–250.00	3.58 ± 0.31
Luteolin 7-O-glucuronide	*y* = 20.132*x* + 20.737	0.9995	1.95–250.00	3.82 ± 0.10
Luteolin-7-O-glucoside	*y* = 17.430*x* − 4.143	0.9999	1.95–250.00	2.21 ± 0.03
Chrysoeriol 7-O-glucuronide	*y* = 17.294*x* − 68.524	0.9996	1.95–250.00	42.09 ± 0.11
Luteolin	*y =* 20.496*x* − 23.782	0.9996	1.95–250.00	1.15 ± 0.04
Chrysoeriol	*y* = 31.792*x* − 4.805	0.9999	1.95–250.00	5.30 ± 0.10
Cyanidin 3-O-glucoside	*y* = 3.678*x* − 18.015	0.9989	1.95–250.00	21.02 ± 0.15
Cyanidin 3-O-(6″-O-malonyl)-glucoside	*y* = 3.861*x −* 33.663	0.9953	1.95–250.00	24.45 ± 0.47
TAC (mg Cyanidin 3-O-glucoside/g DW)	*y* = 3.6783*x* − 18.015	0.9989	3.91–250	55.39 ± 1.53
TPC (mg Galic acids/g DW)	*y* = 0.009*x* + 0.0402	0.9975	3.19–102	242.26 ± 1.86
TFC (mg Rutin/g DW)	*y* = 0.0004*x* + 0.019	0.9999	130–1820	138.80 ± 1.65

### 3.2. Glucose Homeostasis

**Figure 1:**
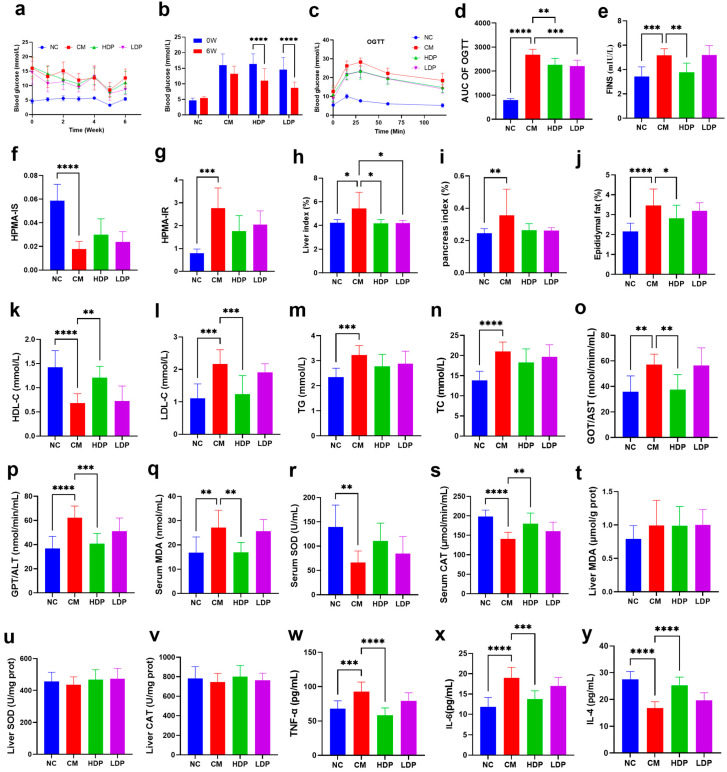
HBP-attenuated STZ-induced diabetes-related symptoms (*n* = 7). (**a**) FBG. (**b**) FBG at 0 and 6 weeks. (**c**) OGTT. (**d**) AUC of OGTT. (**e**) FINS. (**f**) HPMA-IS. (**g**) HPMA-IR. (**h**) Liver index. (**i**) Pancreas index. (**j**) Epididymal fat index. (**k**) HDL-C. (**l**) LDL-C. (**m**) TG. (**n**) TC. (**o**) GOT/AST. (**p**) GPT/ALT. (**q**) Serum MDA. (**r**) Serum SOD. (**s**) Serum CAT. (**t**) Liver MDA. (**u**) Liver SOD. (**v**) Liver CAT. (**w**) TNF-a. (**x**) IL-6. (**y**) IL-4. ANOVA was performed to analyze statistical differences. * *p* < 0.05, ** *p* < 0.01, *** *p *< 0.001, **** *p* < 0.0001. Note: CM means diabetic control group; NC means normal control group; HDP means diabetic with high-dose HBP group; LDP means diabetic with low-dose HBP group, the same in Figure 2, Figure 3, Figure 4 and Figure 5.

The fasting blood glucose (FBG) levels are illustrated in Figure 1a,b. Initially, the CM and HBP administration groups exhibited similar FBG levels, which were significantly higher than those in the NC group. The FBG levels in the HBP administration groups exhibited a progressive decline from week 1 to week 6. When comparing the FBG levels at week 6 to those at week 0, the FBG levels in the HBP groups were markedly lower at week 6 (*p* < 0.05, Figure 1b). This indicates that HBP can significantly reduce the FBG levels in diabetic mice.

The glucose tolerance test (OGTT) results for high blood pressure (HBP) in diabetic mice are presented in Figure 1c,d. Blood glucose levels in diabetic mice increased within 30 min following oral glucose gavage; however, the HBP administration groups exhibited a reduction in blood glucose levels (vs. CM group). After 120 min of oral glucose gavage, the blood glucose levels in both the HDP and LDP groups were significantly lower than those of the CM group (*p* < 0.01, Figure 1d). These findings suggest that HBP can significantly ameliorate glucose intolerance in diabetic mice.

Figure 1e illustrates a significant increase in fasting insulin (FINS) levels in the CM group (vs. NC group), indicating the development of insulin resistance in type 2 diabetic mice. This upward trend was notably improved in the HDP group (vs. CM, *p* < 0.01). Furthermore, Figure 1f shows that the homeostasis model assessment of insulin sensitivity (HPMA-IS) significantly decreased in the CM group (vs. NC group). However, there was no significant increase in the HPMA-IS values in the HBP administration groups compared to the CM group (*p* > 0.05). Additionally, the homeostasis model assessment of insulin resistance (HPMA-IR) was significantly elevated in the CM group (vs. NC, Figure 1g), but HBP administration did not improve this upward trend in the HDP and LDP groups (*p* > 0.05).

### 3.3. Detection of Organ Index

To estimate lipid accumulation in the organs of T2DM mice, we assessed the HBP effects on organ indexes, specifically the liver, pancreas, and epididymal fat. The organ indexes for the liver, pancreas and epididymal fat of the CM group were significantly increased compared with the NC group (Figure 1h–j, *p* < 0.05). In contrast, the liver and epididymal fat indexes of the HDP group were significantly decreased compared to the CM group (*p* < 0.05). The results suggest that HBP exerts a protective effect on the liver and epididymal fat in mice.

### 3.4. Serum Biochemical Parameters

Dyslipidemia is one of the most serious risk factors for T2DM. As shown in Figure 1k–n, the levels of HDL-C were significantly decreased, while the levels of LDL-C, TG and TC were dramatically increased in the CM group compared to the NC group (*p* < 0.001). In contrast, the HDL-C levels were significantly increased, and the levels of LDL-C were markedly reduced in the HDP group (vs. CM group, *p* < 0.01). This suggests that high-concentration HBP administration could effectively alleviate dyslipidemia, as evidenced by the normalization of HDL-C and LDL-C levels. Serum AST and ALT, recognized as specific indicators of liver damage, are presented in Figure 1o,p. As shown in the figure, the serum levels of AST and ALT in the CM group were significantly increased (vs. NC group, *p* < 0.01). In contrast, the HDP group showed a dramatic decrease in AST and ALT levels (vs. CM group, *p* < 0.01). This indicates that high-concentration HBP administration has a protective role in liver function.

### 3.5. Antioxidant Assay

The HBP effects on the antioxidant levels in serum and liver were evaluated, as illustrated in Figure 1q–v. Compared with the NC group, the CM group showed significantly increased serum MDA levels and decreased SOD and CAT activities (*p* < 0.01). Conversely, the HDP group showed a significant reduction in MDA levels and an increase in CAT activity when compared to the CM group (*p* < 0.01). A similar trend was observed in liver antioxidants; however, the observed effects did not reach statistical significance (*p* > 0.05). These results suggest that high concentrations of HBP administration have positive effects on serum antioxidant capacity.

### 3.6. Inflammatory Factors

To evaluate the impact of HBP on inflammatory cytokines, the serum levels of TNF-α, IL-4, and IL-6 were assessed using the reagent test kits (Figure 1w–y). The levels of TNF-α and IL-6 of the CM group were dramatically increased (vs. NC group, *p* < 0.001), while the level of IL-4 was significantly decreased (*p* < 0.0001). In the HDP and LDP groups, TNF-α and IL-6 levels were dramatically reduced (*p* < 0.001), and the level of IL-4 was significantly increased (*p* < 0.0001), compared to the CM group. These findings suggest that HBP administration can significantly improve the inflammatory condition in mice with T2DM.

### 3.7. Histopathology

**Figure 2:**
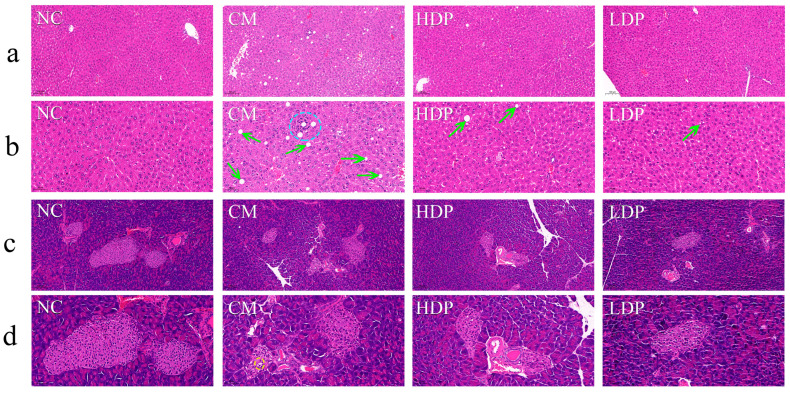
Histopathological analysis of the liver and pancreas in different groups of mice via HE staining. Liver tissue sections: (**a**) ×200, (**b**) ×400, blue circle in CM group: inflammatory cell infiltration within the liver lobule, green arrow in CM, HDP and LDP groups: cells hepatic steatosis; Pancreas tissue sections: (**c**) ×200, (**d**) ×40, yellow circle in CM group: β-cell swelling and vacuolization.

In the NC group, the liver lobules exhibited a normal structure, with clearly defined cell outlines arranged regularly like corn kernels, and no obvious lesions (Figure 2a,b). In contrast, the CM group displayed hepatic steatosis, focal necrosis and inflammatory cell infiltration within the liver lobule cells. Compared to the CM group, the administration of HBP significantly alleviated these pathological changes in the liver. The structure of pancreatic acini and islets appeared normal in the NC group, with clearly defined islet cells arranged in an orderly manner (Figure 2c,d). In comparison to the NC group, the CM group showed a decrease in the number of islet cells, with a disordered and scattered arrangement, as well as enlarged cell volumes forming bubbles. However, in the HBP administration groups, the number of islet cells increased, and the aforementioned pathological changes in the pancreas were alleviated.

### 3.8. Gut Microbiota and SCFAs

#### 3.8.1. Community Diversity Analysis

**Figure 3:**
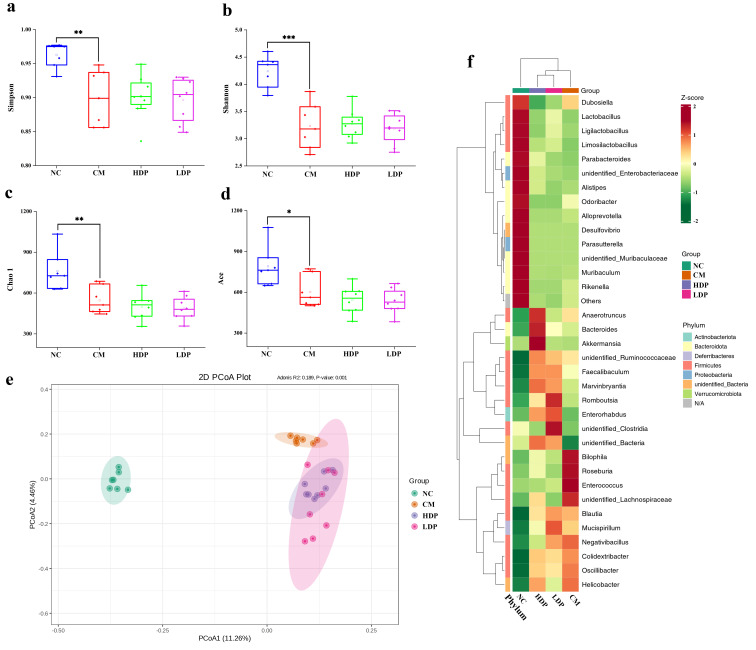
Effects of HPB on alpha diversity and beta diversity. (**a**) Simpson index, (**b**) Shannon index, (**c**) Chao1 index, (**d**) Ace index. (**e**) PCoA. (**f**) HCA. ANOVA was performed to analyze statistical differences. * *p* < 0.05, ** *p* < 0.01, *** *p* < 0.001.

The diversity of the gut microbiota community was evaluated via α diversity and β diversity. As shown in Figure 3a–d, the Shannon, Simpson, Ace, and Chao1 indices of the fecal samples of the CM group significantly declined compared with NC group (*p* < 0.05). However, these four indices in the HDP and LDP groups did not show notable changes compared to the CM group (*p* ≥ 0.05), suggesting that HPB administration does not enhance the diversity and abundance of gut microbiota in diabetic mice. Beta diversity reflects differences in the microbial communities among samples. The CM group exhibited a distinct separation from the NC group in terms of microbiota composition, suggesting significant alterations in the gut microbiota of diabetic model mice (Figure 3e). The HPB administration groups tended to cluster closer to the CM group, with all groups deviating from the NC group, suggesting that HPB administration does not mitigate the impact of T2DM on the β diversity of gut microbiota. The PCoA analysis was consistent with the hierarchical clustering results (Figure 3f).

#### 3.8.2. Gut Microbiota Composition

**Figure 4:**
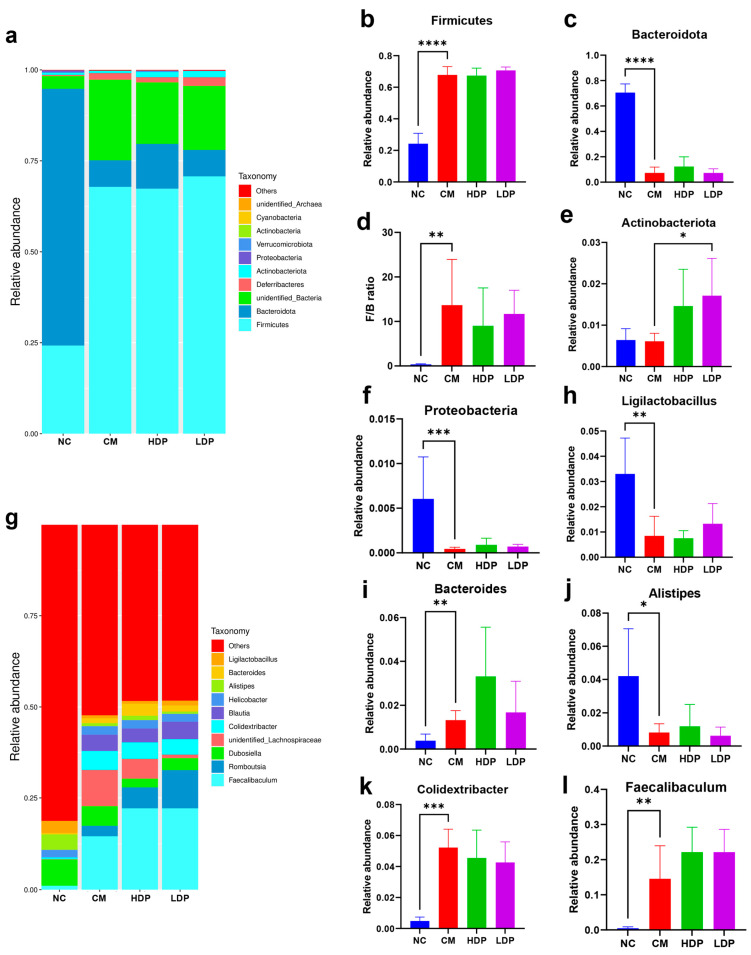
Effects of HPB on gut microbiota composition. (**a**) Gut microbiota composition at the phylum level. (**b**–**f**) Relative abundance of identified differential abundant bacterial group at the phylum level. (**g**) Gut microbiota composition at the genus level. (**h**–**l**) Relative abundance of identified differential abundant bacterial group at genus level. ANOVA was performed to analyze statistical differences. * *p* < 0.05, ** *p* < 0.01, *** *p* < 0.001, **** *p* < 0.0001.

At the phylum level, Firmicutes and Bacteroidota were identified as the main dominant bacteria in the gut microbiota of all four groups (Figure 4a), with their relative abundance accounting for over 75% of the total bacterial population. Compared with the NC group, The ratio of Firmicutes to Bacteroidetes (F/B) and the Firmicutes abundance were dramatically increased (*p* < 0.01, Figure 4b,d), while the abundance of Bacteroidota and Proteobacteria were dramatically reduced in the CM group (*p* < 0.001, Figure 4c,f). In comparison with the CM group, the relative abundance of Bacteroidota and Proteobacteria exhibited an upward trend in both the HDP and LDP groups, although the differences were not statistically significant (*p* ≥ 0.05, Figure 4c,f). Additionally, the Actinobacteria abundance in LDP groups was significantly higher compared to the CM group (*p* <0.05, Figure 4e). At the genus level (Figure 4g), in the HBP administration groups, Faecalibaculum, Romboutsia, Dubosiella, Colidextribacter, and Blautia were identified as the predominant bacteria genera. The relative abundance of Ligilactobacillus and Alistipes was significantly reduced compared with the NC group (*p* < 0.05, Figure 4h,j), while the relative abundance of Bacteroides and Colidextribacter saw a drastic increase in the CM group (*p* < 0.05, Figure 4i,k). In comparison to the CM group, HBP administration resulted in an increased relative abundance of Lactobacillus, Alistipes, and Faecalibaculum, and a decrease in the relative abundance of Colidextribacter; however, these changes did not reach statistical significance (*p* > 0.05, Figure 4h–k). These findings indicate that the alterations in gut microbiota were primarily driven by the CM group, and the intervention of HBP showed minimal modulation of microbial diversity and composition.

#### 3.8.3. SCFAs

The contents of SCFAs, AA, PA, IVA, BA and IBA were significantly downloaded into the CM group compared with the NC group, (*p* < 0.01, Supplementary Figure S2). These results indicated the T2DM could download the contents of short-chain fatty acids in mice feces. However, the contents of SCFAs and six short-chain fatty acids in HBP administration groups were not significantly different compared with the CM group.

### 3.9. Hepatic Transcriptome

**Figure 5:**
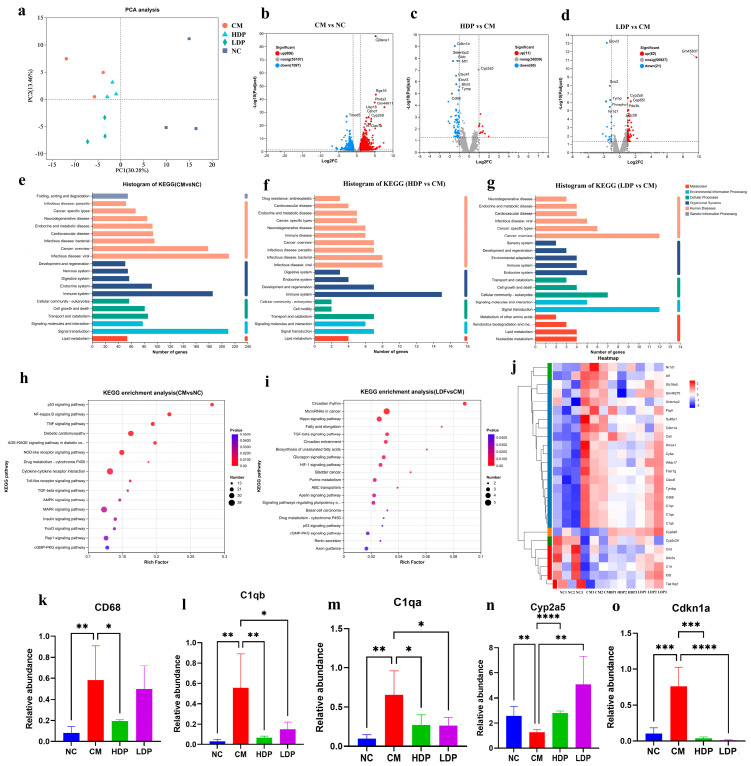
Transcriptomics. (**a**) PCA plot, (**b**–**d**) Volcano plot, (**e**–**g**) KEGG function annotation analysis, (**h**,**i**) KEGG functional enrichment analysis, (**j**) Heatmap analysis of DEGs with large expression differences, (**k**–**o**) qRT-PCR analysis of CD68, C1qb, C1qa, Cyp2a5 and Cdkn1a mRNA expression. ANOVA was performed to analyze statistical differences. * *p* < 0.05, ** *p* < 0.01, *** *p* < 0.001, **** *p* < 0.0001.

To investigate the functions of HBP in the molecular mechanisms underlying hepatic lesions, we conducted RNA sequencing of liver tissues. A total of 33,078 expressed genes were identified in this analysis, with the percentage of Q30 bases exceeding 94.64%. PCA analysis demonstrated distinct segregation of liver transcript abundance among the NC, CM, HDP, and LDP groups (Figure 5a). Using adjusted *p*-values (*p*-adjust < 0.05) and a fold-change threshold (>2) as screening criteria, we found that 1097 genes were significantly down-regulated, while 806 genes were markedly up-regulated in the CM group compared to the NC group (Figure 5b). In the HDP group, we identified 60 down-regulated genes and 11 up-regulated genes, while the LDP group exhibited 21 down-regulated genes and 62 up-regulated genes compared to the CM group (Figure 5c,d). KEGG gene function annotation analysis of the differentially expressed genes (DEGs) between the CM and NC groups revealed that the enriched signaling pathways were associated with infectious diseases, signal transduction and the immune system (Figure 5e). The DEGs of the HDP group (vs. CM group) are annotated in the immune system, signal transduction, transport and catabolism (Figure 5f), while those in the LDP group (vs. CM group) encompassed cancer: overview, signal transduction, and cellular community-eukaryotes (Figure 5g). Further analysis of functional enrichment in KEGG pathways is illustrated in Figure 5h,i. In the comparison of CM vs. NC, the bubble plot displays only the top 16 signaling pathways associated with T2DM, including the p53 signaling pathway, NF-kappa B signaling pathway, TNF signaling pathway, and AGE-RAGE signaling pathway relevant to diabetic complications (Figure 5h). Additionally, the top 20 pathways with the highest enrichment levels were presented for the HBP administration groups. In the comparison of HDP vs. CM (Supplementary Figure S3), pathways related to cell adhesion molecules, which are part of signal transduction, and pathways such as leukocyte transendothelial migration, complement and coagulation cascades, and neutrophil extracellular trap formation, which are associated with the immune system, may be linked to T2DM. In the comparison of LDP vs. CM (Figure 5i), pathways associated with T2DM were observed, including the Hippo signaling pathway, TGF-beta signaling pathway, HIF-1 signaling pathway, drug metabolism via cytochrome P450, p53 signaling pathway, and cGMP-PKG signaling pathway. Furthermore, numerous differentially expressed genes (DEGs) with an average expression level greater than 10, from the comparisons CM vs. NC, HDP vs. CM, and LDP vs. CM, were selected to create a heat map (Figure 5j). To validate the results of the RNA-seq analysis, a total of five DEGs, namely CD68, C1qa, C1qb, Cyp2a5, and CDKN1A were selected for qRT-PCR analysis. The qRT-PCR expression findings of these genes were consistent with the RNA-seq analysis (Figure 5k–o), showing four genes with increased expression (CD68, C1qa, C1qb, and CDKN1A) and one gene with decreased expression (Cyp2a5) in the HBP administration groups compared with the T2DM group. Furthermore, in the analysis of DEGs from the immune system and signal transduction pathways (Supplementary Figure S4), CDKN1A was found to interact with Sox9 and Serpine1, which are involved in T2DM-related signaling pathways, including cAMP, HIF-1, p53, Hippo, Apelin signaling pathways, and the AGE-RAGE signaling pathway in diabetic complications.

## 4. Discussion

Hulless barley brans are typically discarded as waste or utilized as low-value animal feed. However, increasing research indicates that hulless barley bran is rich in bioactive compounds. Hulless barley bran contains diverse phenolic compounds, mainly phenolic acids (hydroxycinnamic acids and hydroxybenzoic acids) and flavonoids (flavonols, flavones, flavanols, flavanonols, and anthocyanidins) [14,15,22]. In the present study, 27 compounds were identified in HBP, including one phenolic acid (protocatechuic acid), 17 flavonoids, and nine anthocyanidins. The predominant flavonoids included derivatives of catechin, quercetin, chrysoeriol, and luteolin, while all nine anthocyanidins were identified as cyanidin derivatives. The sugars present were glucoside, galactoside, and glucuronide. The TAC, TPC and TFC in the fractions were measured at 55.39, 242.336 and 138.8 mg/g, respectively. These results are consistent with previous study findings that hulless barley is a rich source of phenolic compounds [9,23]. Additionally, in our previous study [24], the high levels of chrysoeriol 7-O-glucuronide (42.09 mg/g), cyanidin 3-O-(6″-O-malonyl)-glucoside (24.45 mg/g), and cyanidin 3-O-glucoside (21.02 mg/g) were quantified in HBP. Polyphenols are characterized by their molecular scaffold, which consists of multiple phenolic rings and hydroxyl functional groups. This structure endows their anti-diabetic effects by inhibiting cytotoxic human amylin aggregation, and alleviating oxidative stress and inflammation, and modulating other pathways such as β-cell-protective and insulin-sensitizing [25]. And Cyanidin-3-O-glucoside enhances insulin sensitivity by up-regulating the expression of GLUT-1 and GLUT-4, and modulating the PI3K/Akt pathway [26]. It was in a significantly dose-dependent manner that the HDP (400 mg/kg BW HBP) group showed a better effect on alleviating lesions rather the LDP (100 mg/kg BW HBP) group, exhibited on physiological and biochemical data biochemical data (Figure 1e,j–l,o–q,s,w–y). So, it is speculated that the abundance of phenolic compounds, particularly anthocyanins and flavonoids, might exert hypoglycemic activity via improvement insulin resistance, enhancement serum antioxidant enzyme activities and modulation inflammatory cytokines.

T2DM is a disorder characterized by impaired blood glucose regulation, resulting in hyperglycemia, insulin deficiency, insulin resistance, and pancreatic amyloid deposition, often accompanied by dyslipidemia and prolonged inflammation [25,27]. Consequently, alleviating these symptoms has become a fundamental indicator for evaluating the hypoglycemic effects of supplements for T2DM [28,29]. In the present study, the CM group exhibited higher levels of FBG, blood glucose, and serum insulin compared to the NC group, consistent with STZ mice models that show elevated blood glucose and insulin levels relative to controls [29]. After six weeks of HBP administration, the elevated FBG, oral glucose tolerance test (OGTT), blood glucose, and serum insulin levels were significantly reduced in the HDP group. Additionally, HBP administration mitigated the increase in organ indices of the liver and epididymal fat, suggesting an improvement in the pathological changes of diabetic mice.

Dyslipidemia, a primary risk factor for cardiovascular disease, frequently coexists with T2DM [30]. Administration of high concentration HBP has been shown to effectively reduce dyslipidemia by normalizing the levels of HDL-C and LDL-C. Serum AST and ALT serve as specific indicators of liver damage [31]. The administration of HBP significantly decreased the levels of AST and ALT compared to those in the control group, indicating that HBP plays a protective role in liver function.

Oxidative stress, involved in the development of insulin resistance, plays an important role in the pathology of diabetes [32,33]. In Figure 1q–s, the CM group exhibited a significant elevation in serum MDA levels along with a marked reduction in SOD and CAT activities (vs. NC group). However, high concentrations of HBP administration positively affected the activities of SOD and CAT in serum, while also reducing serum MDA content. These findings suggest that HBP may stimulate the oxidative stress defense mechanisms and mitigate oxidative stress in vivo. Polyphenols possess the antioxidant properties and capacity to protect cells against oxidative stresses that are linked to scavenge ROS, reduce lipid peroxidation, increase antioxidant enzyme activity, inhibit the mitogenactivated protein kinase (MAPK)/JNK pathway that ediates apoptosis in β-cells [34], and chelate metal ions [25].

Pro-inflammatory cytokines serve as an intermediary in the immune response, exerting toxic effects on pancreatic β-cells, and activating the inflammatory cytokine signaling pathways [35]. In this study, the levels of pro-inflammatory cytokines TNF-α and IL-6 were down-regulated, while the anti-inflammatory cytokine IL-4 was up-regulated under HBP treatment. This suggests that HBP may have the potential to alleviate the onset and progression of diabetes. Numerous studies indicate that inhibiting inflammatory factor release could ameliorate insulin signaling and reduce hyperglycemia [27,36].

The liver plays a crucial role in maintaining blood glucose balance by producing glucose through glycogen degradation and gluconeogenesis, as well as storing glucose as glycogen [37]. The pancreas is also essential in regulating blood glucose levels via the islets of Langerhans, which secrete glucagon, insulin, somatostatin, and ghrelin hormones [38]. In the present study, disarrangement of hepatocytes, inflammatory cell infiltration and necrotic cells were observed in the livers of the T2DM-group mice (Figure 2a,b), consistent with previous findings [39]. Additionally, the injuries to the pancreas in streptozotocin-induced T2DM mice were noted here (Figure 2c,d), including islet shrinkage, irregular islet morphology, cellular swelling, cell vacuolation, and apoptosis, which align with previously reported studies [40]. The HBP administration groups significantly alleviated the pathological changes of hepatic steatosis, focal necrosis and inflammatory cell infiltration in the liver, while also increasing the number of islet cells and mitigating the pathological changes in the pancreas.

Gut microbes play a fundamental role in human nutrition and metabolism [41,42]. Alterations in microbiota diversity, composition, and function may be associated with metabolic disorders, including T2DM and its related conditions [41,43,44,45]. In the present study, the alpha diversity indices, including Shannon, Simpson, Ace, and Chao1, of T2DM model mice were significantly reduced compared to normal mice. This finding is consistent with previous reports demonstrating an association between lower bacterial diversity and disease conditions [46,47]. Gaike, Paul, Bhute, Dhotre, Pande, Upadhyaya, Reddy, Sampath, Ghosh, Chandraprabha, Acharya, Banerjee, Sinkar, Ghaskadbi, Shouche and Whiteson [45] revealed that the gut microbial abundance and diversity of diabetics differed significantly from those of nondiabetics and tended to recover toward that of nondiabetics following anti-diabetic treatment. However, α diversity did not show significant differences compared to diabetic model mice, nor did it recover to normal levels after HBP administration. Similarly, the β diversity in the HBP administration groups was distinct from the NC group and was closer to the CM group. Song et al. [48] also found that the diversity and richness of microbiota in HFD-induced obese C57BL/6J mice were not altered by anthocyanin extract from black rice. These results imply that HBP administration may not alleviate the damage of T2DM by increasing the diversity of gut microbiota.

The composition of gut microbiota was analyzed to evaluate the alleviating effects of HBP on diabetes. At the phylum level, the relative abundances of Firmicutes and Bacteroidota accounted for 97.49%, 75.15%, 79.64%, and 77.99% of the total bacterial population in the NC, CM, HDP and LDP groups, respectively. The gut microbial communities are primarily composed of Firmicutes and Bacteroidetes [49,50,51], which convert indigestible carbohydrates into short-chain fatty acids (SCFAs) such as butyrate, propionate, and acetate [51,52,53]. Additionally, the elevation of the F/B ratio has been recognized as a marker of intestinal microbiota imbalance [51,54]. In the present study, HBP administration decreased the elevated F/B ratio, although this did not reach statistical significance (*p* ≥ 0.05). Furthermore, the administration of HBP significantly increased Actinobacteria relative abundance in the LDP group (*p* < 0.05) and slightly elevated Proteobacteria relative abundance in the HBP administration groups (*p* ≥ 0.05) compared to the CM group. At the genus level, a reversion effect was observed in the relative abundances of Ligilactobacillus, Bacteroides alistipes, and Feacalibaculum in the HBP administration groups compared to the CM group, although this did not reach statistical significance. However, minimal modulation of microbial composition was observed in the present study.

Short-chain fatty acids (SCFAs), the primary end-products of dietary fiber fermentation by gut microbiota, can bind to G protein-coupled receptor (GPCR) to stimulate the intestinal secretion of glucagon-like peptide-1 (GLP-1) and peptide YY (PYY) [42]. This process subsequently increases energy expenditure [55], reduces food intake [56], and enhances glucose metabolism and insulin secretion [57,58,59]. Consistent findings indicate that lower fecal SCFA concentrations are observed in individuals with T2DM compared to controls [41,42,51,60]. Thus, increasing SCFA levels may represent a viable strategy for diabetes treatment [61,62]. However, the beneficial effects of elevating SCFA levels, including AA, PA, IVA, BA, IBA and VA, have not been investigated in groups administered with HBP. These results may be related to the reduction of relative abundance of Firmicutes and Bacteroidetes (compared to normal mice), which are known to produce SCFAs [51,52,53].

In the present study, the DEGs obtained from the comparisons between the CM and NC groups, as well as the HBP administration group and CM, were enriched in signal transduction and immune system pathways (Figure 5e–g). Similar results have been reported in previous studies [63,64]. Furthermore, signaling pathways, such as the p53 signaling pathway, NF-kappa B signaling pathway, TNF signaling pathway, AGE-RAGE signaling pathway, and Foxo signaling pathway, were observed in the comparison between CM and NC (Figure 5h). These signaling pathways were significantly affected by T2DM [65,66,67,68,69]. Under HBP administration, the enriched DEGs included pathways such as the Hippo signaling pathway, TGF-beta signaling pathway, HIF-1 signaling pathway, p53 signaling pathway, and cGMP-PKG signaling pathway, which have also been reported in T2DM research [69,70,71,72,73]. Among the five validation genes, CD68, C1qb, and C1qa were involved in the immune system, while CDKN1A was associated with multiple signal transduction pathways in the present study. CD68, which is related to the lysosomal-associated membrane protein (LAMP) family, is recognized as a monocyte/macrophage marker [74]. High expression levels of CD68 were observed in the CM group, indicating that these mice were in a hyper-inflammatory state. Zampieri, Karpach, Salerno, Raguzzini, Barchetta, Cimini, Dule, De Matteis, Zardo, Borro, Peluso, Cavallo and Reale [63] also reported elevated CD68 expression in T2DM patients. C1qb and C1qa are part of the C1qDC gene family and play a significant role in the immune responses of grass carp [75]. Additionally, C1qb has been identified as a candidate gene for diabetes susceptibility located on mouse chromosome 4 [76]. C1qb has the potential to increase the number of macrophages, leading to pancreatic islet β-cell damage and promoting T1DM in rats [77]. In the present study, HBP administration significantly reduced the elevated expressions of CD6, C1qb and C1qa, suggesting that HBP may alleviate symptoms of T2DM by enhancing immune responses, which aligns with the favorable results observed for inflammatory factors (Figure 1w–y). Furthermore, compared to the NC group, the expression of CDKN1A was significantly increased in the CM group. CDKN1A, a cyclin-dependent kinase inhibitor-1, is associated with p53/tp53-mediated inhibition of cellular proliferation in response to DNA damage [78]. The overexpression of cyclin-dependent kinase inhibitors regulates controls the progression of the cell cycle into the G1phase, thereby reducing cell proliferation in clonal B-cells [79]. Muhamma, et al. [80] also reported that abnormal expression of CDKN1A impairs impaired the secretion of insulin and glucagon in clonal β- and α-cells. However, HBP was able to down-regulate the overexpression of CDKN1A, which interacts with other T2DM-related genes, such as Sox9 and Serpine1, and is responsible for modulating signaling pathways, including HIF-1, FoxO, p53, PI3K-Akt, and Jak-STAT. These pathways are associated with glucose uptake, insulin resistance, and the adipocytokine pathway contributing to the pathogenesis of T2DM [80].

## 5. Conclusions

Hulless barley brans are typically discarded as waste or utilized as low-value animal feed. In this study, 27 polyphenols were identified in the polyphenolic composition of black hulless barley bran (HBP) using UPLC-QTOF-MS/MS. The HBP exhibited a TAC of 55.39 mg/g, a TPC of 242.336 mg/g, and a TFC of 138.8 mg/g. Furthermore, high concentrations of Chrysoeriol 7-O-glucuronide (42.09 mg/g), Cyanidin 3-O-glucoside (21.02 mg/g), and Cyanidin 3-O-(6′′-O-malonyl)-glucoside (24.45 mg/g) were quantified in HBP using UPLC.

The results from the animal tests indicate that HBP can effectively alleviate the conditions of T2DM mice induced by STZ. Specifically, it reduces FBG, lowers the liver index, improves glucose intolerance, alleviates dyslipidemia, enhances serum antioxidant capacity, reduces inflammation, and mitigates liver and pancreatic injury. However, HBP had minimal effects on regulating the diversity and abundance of the gut microbiota and SCFA. Furthermore, analysis of the mouse hepatic transcriptome revealed that the DEGs (CM vs. NC, HDP vs. CM) were enriched in signal transduction and the immune system. More specifically, HBP administration influenced the expression of genes associated with diabetes, such as CDKN1A, CD68, C1qa, and C1qb, which are linked to signaling pathways involving Hippo, TGF-beta, HIF-1, p53, and cGMP-PKG. These findings suggest that HBP may modulate these signaling pathways to regulate blood glucose levels and insulin secretion.

These findings could provide a scientific basis for the research and development of functional foods and dietary supplements derived from black hulless barley bran.

## Data Availability

The original contributions presented in the study are included in the article/Supplementary Material, further inquiries can be directed to the corresponding author.

## Supplementary Materials

The following supporting information can be downloaded at: https://www.mdpi.com/article/10.3390/foods14172994/s1, Figure S1: MS spectra of the 27 compounds identified in HBP; Figure S2: The effects of HBP on the contents of SCFAs; Figure S3: KEGG functional enrichment analysis of HDF vs. CM; Figure S4: IPP of Degs from immune system and signaling transduction; Table S1: Feed ratios; Table S2: Primer sequences used for real-time PCR analysis.
