# Supplementary material for: Characteristics of Polyphenols of Black Hulless Barley Bran and Its Anti-Diabetic Activity
Source: Foods. 2025 Aug 27;14(17):2994. doi: 10.3390/foods14172994 (PMC12428599; doi:10.3390/foods14172994)

Supplementary Table S1

Feed ratios

Raw Material	High-fat diet		Normal diet	
	Energy（gm）	calories（kcal）	Energy（gm）	calories（kcal）
Casein	200	800	200	800
L-Cystine	3	12	3	12
Corn Starch	0	0	506.2	2024.8
Maltodextrin 10	125	500	125	500
Sucrose	72.8	291.2	72.8	291.2
Cellulose, BW200	50	0	50	0
Soybean Oil	25	225	25	225
Lard	245	2205	20	180
Mineral Mix S10026B	50	0	50	0
Vitamin Mix V10001C	1	4	1	4
Choline Bitartrate	2	0	2	0
FD&C Red Dye #40	0	0	0	0
FD&C Yellow Dye #5	0	0	0.04	0
FD&C Blue Dye #1	0.05	0	0.01	0
Total	773.85	4037.2	1055.05	4037

Supplementary Table S2

Primer sequences used for real-time PCR analysis.

Name	Forward primer (5′–3′)	Reverse primer (3′–5′)
CD68	GGGCTCTTGGGAACTAC	ATGAGCCCGAGACTACA
C1qb	CTATGAGCCACGCAACG	GTCTTTCGGTCACTTCTAC
C1qa	TGTGCTGACCATGACCCTA	AACGGACCTAACGGAAA
Cyp2a5	CAAAGACTTCAACCCAA	AAGAGGCTTTGTTATGG
Cyba	GCCATGTGGGCCAACGA	CCTATGAGGTCGTCTGTCT
Cdkn1a	CCGAGAACGGTGGAACT	GTCGTCCCGTCTCCTTC

Supplementary Figure S1

MS spectra of the identified compounds in HBP

[M-H]⁻ (m/z)

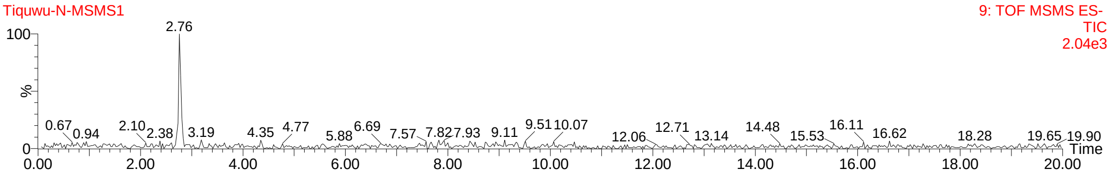

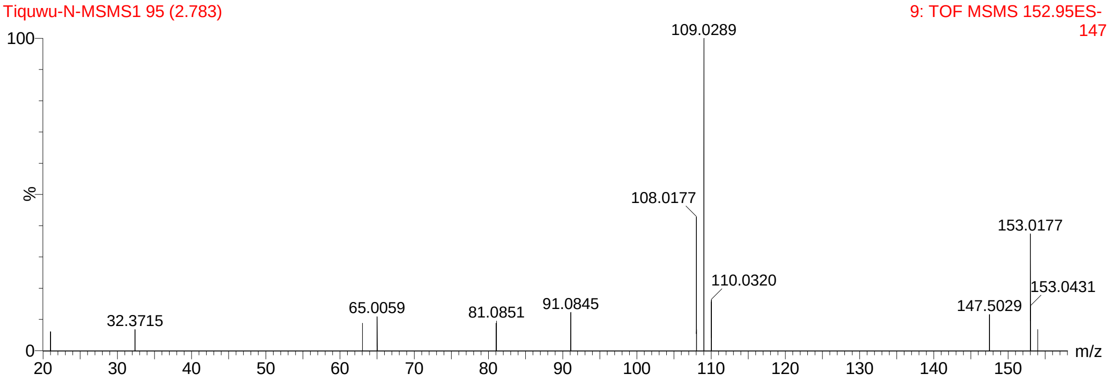

1 Protocatechuic acid

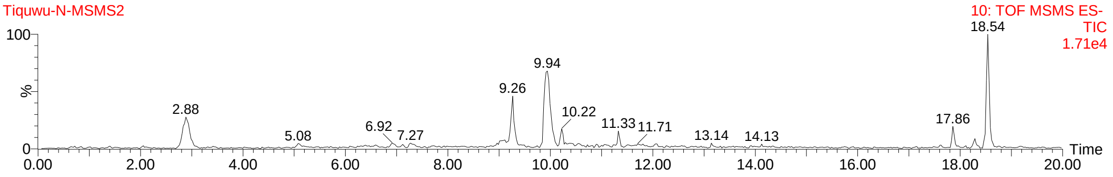

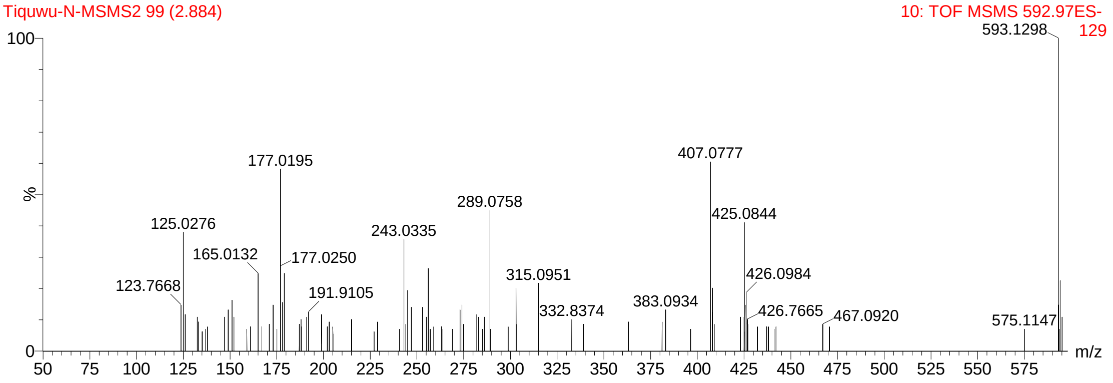

2 (Epi)gallocatechin-(epi)catechin

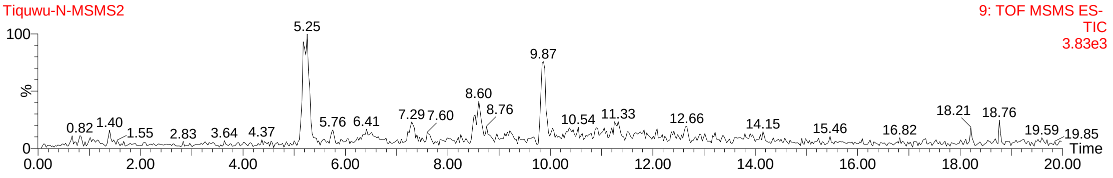

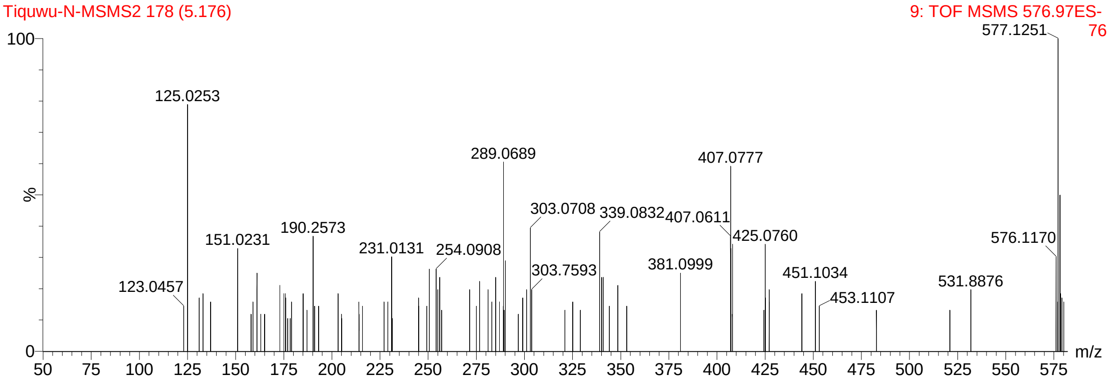

3 (Epi)catechin-(epi)catechin

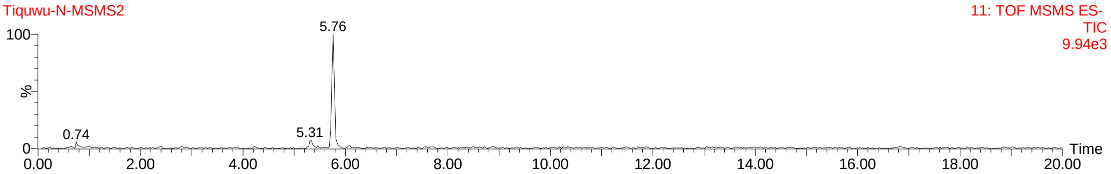

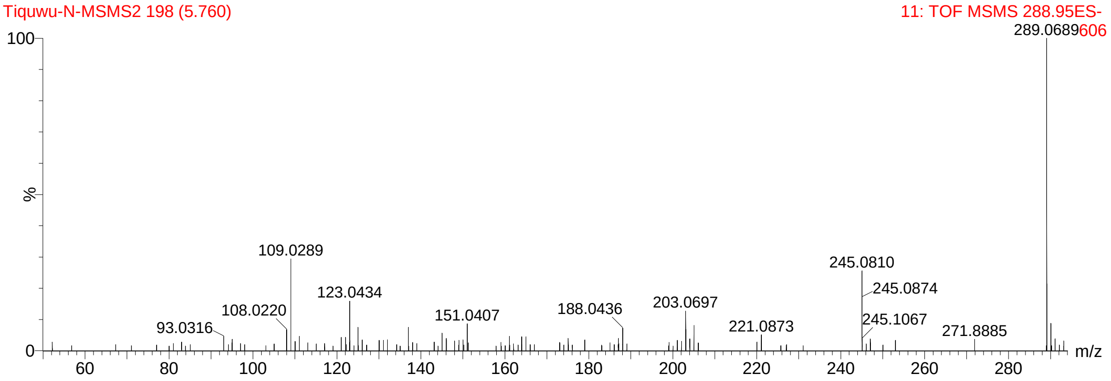

4 Catechin

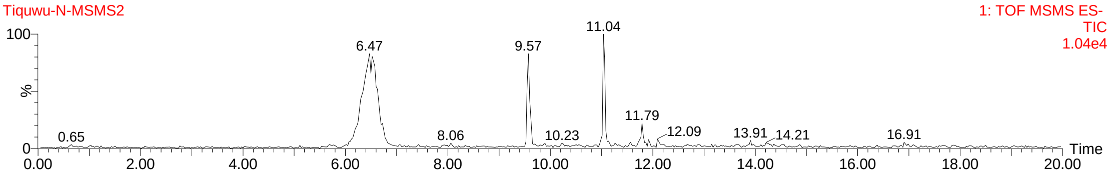

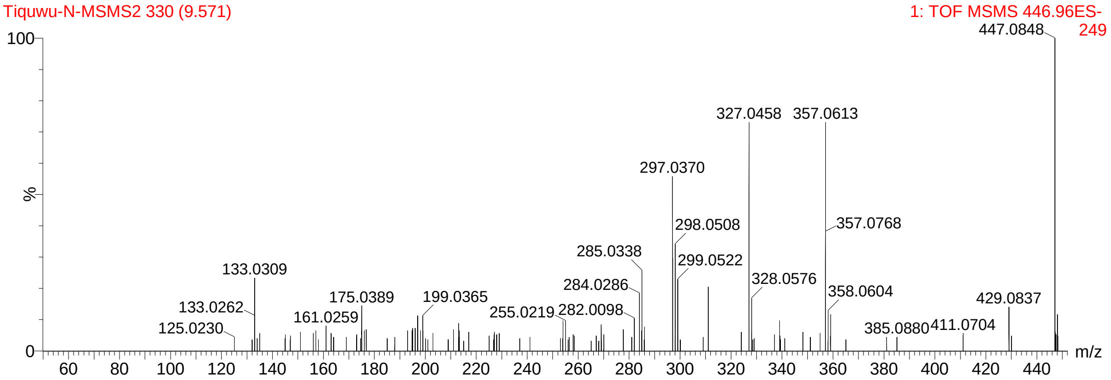

5 Orientin or Isoorientin

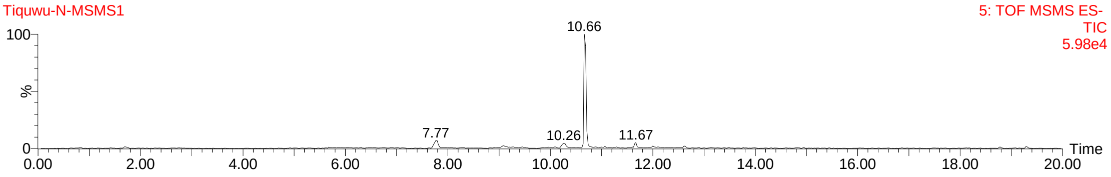

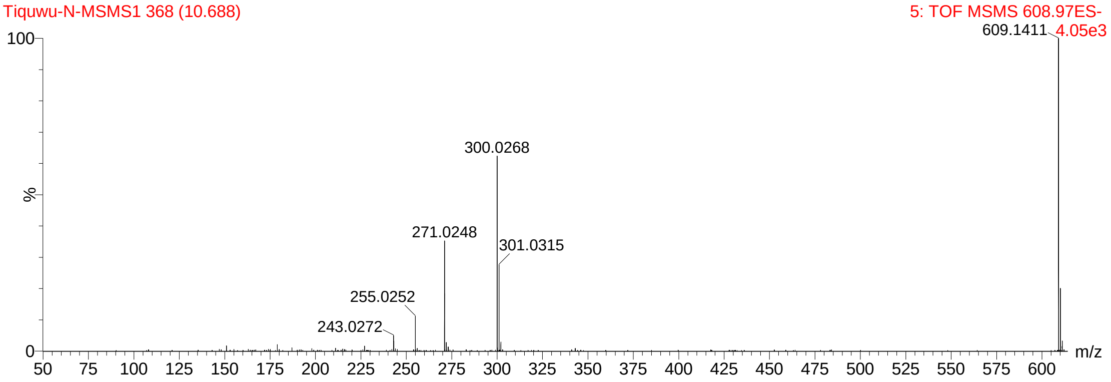

6 Quercetin 3-O-rutinoside

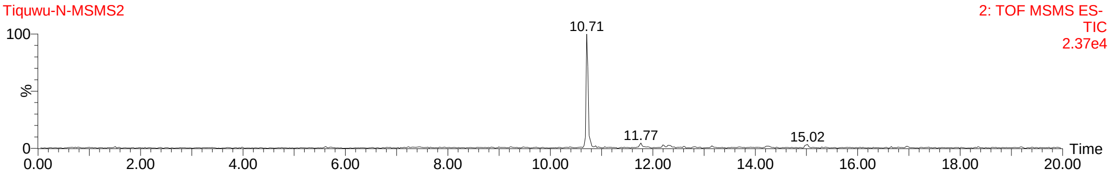

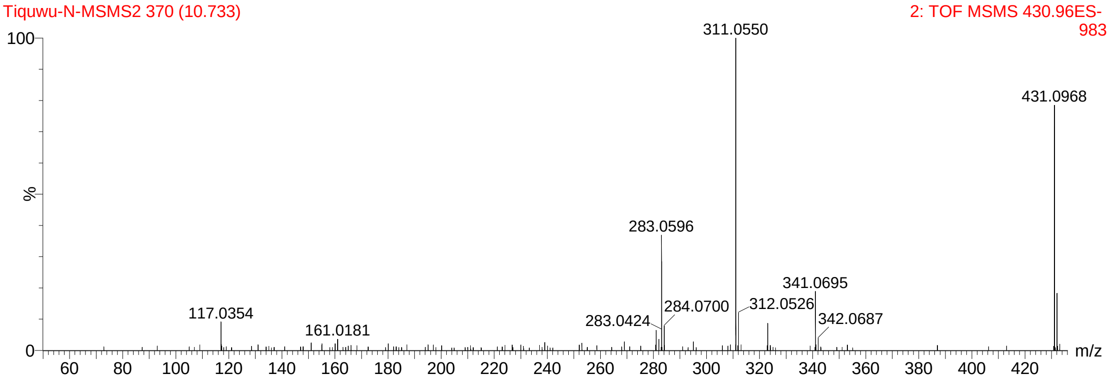

7 Isovitexin or vitexin

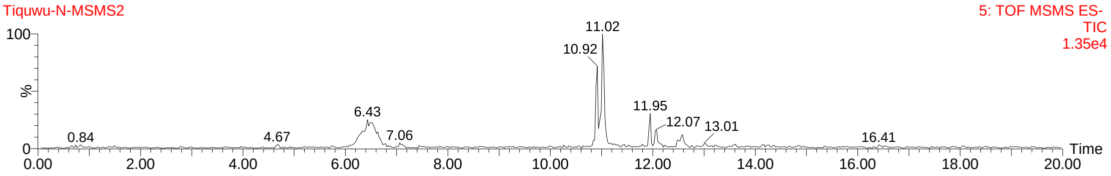

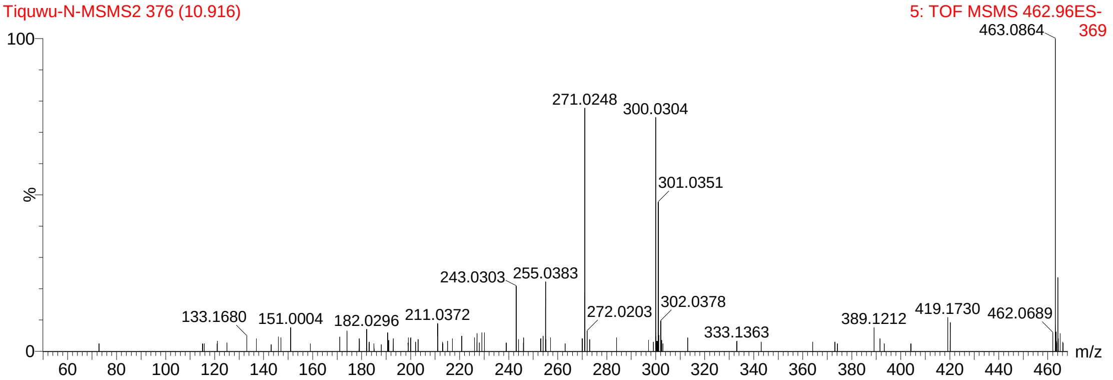

8 Quercetin-3-O-glucoside

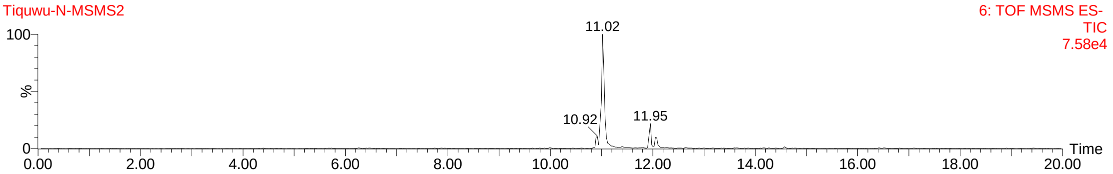

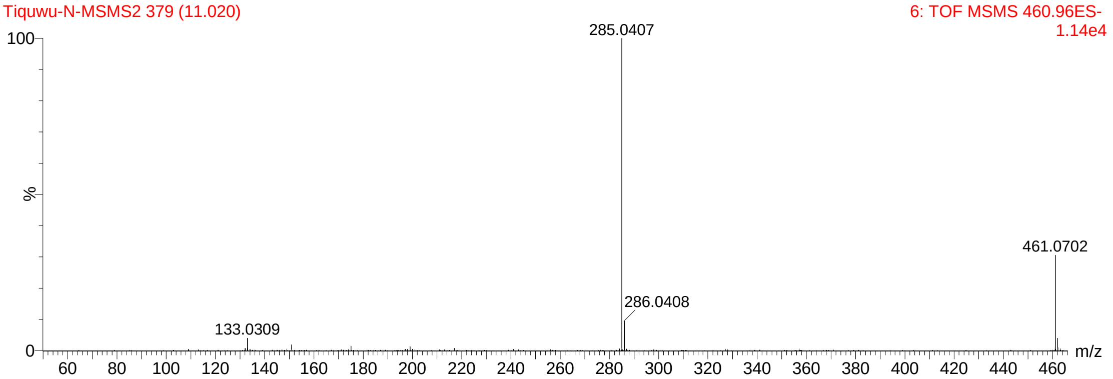

9 Luteolin 7-O-glucuronide

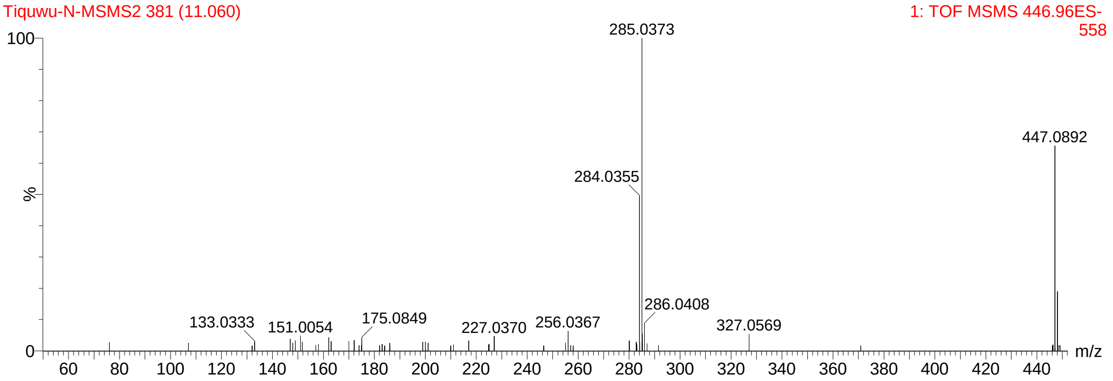

10 Luteolin-7-O-glucoside

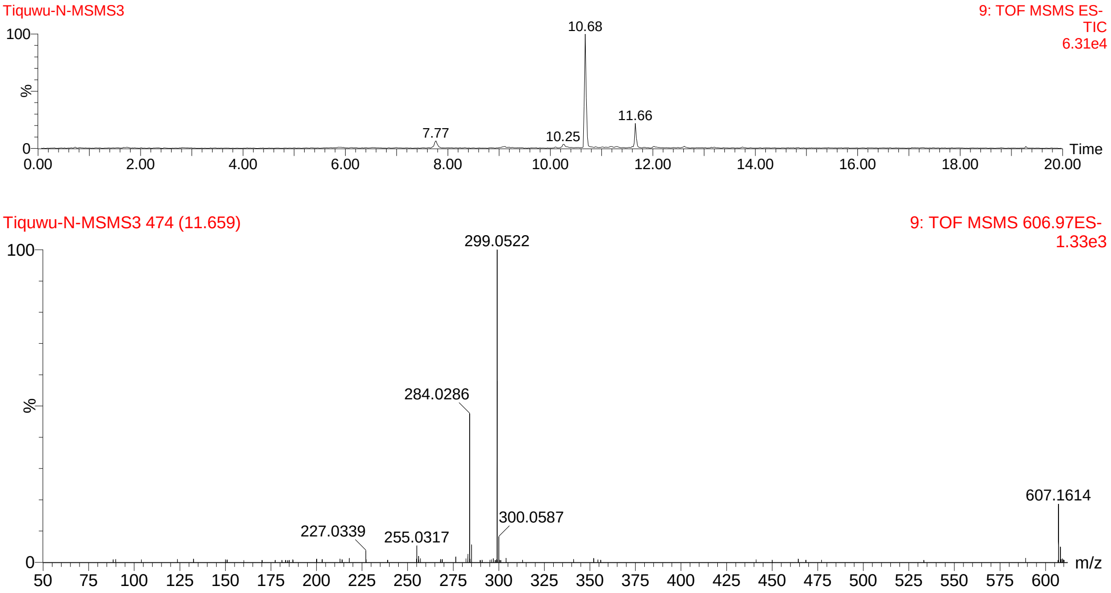

11 Chrysoeriol 7-O-rutinoside

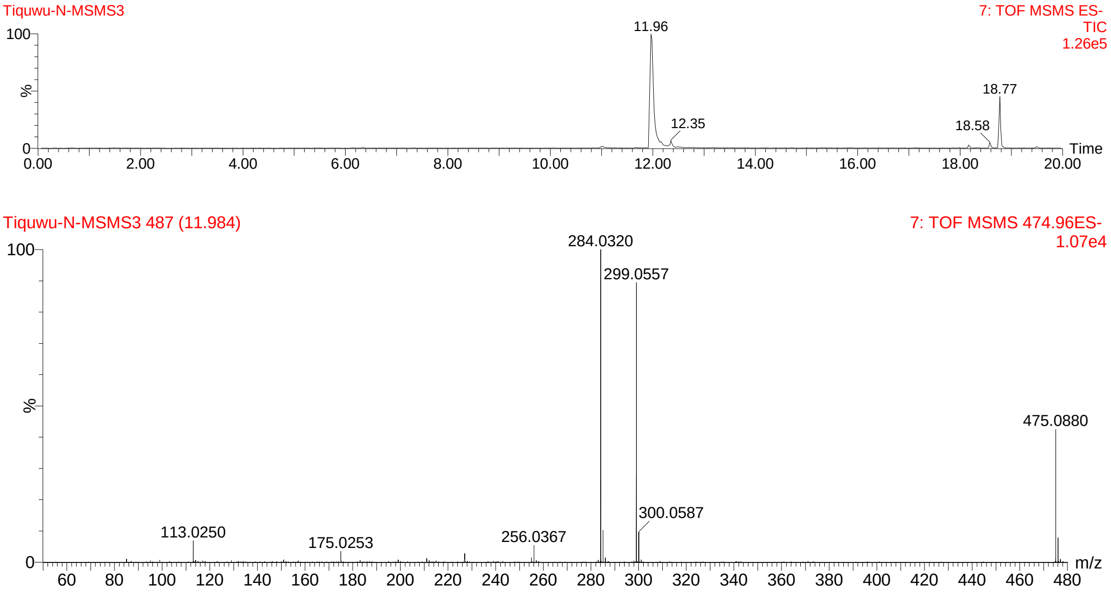

12 Chrysoeriol 7-O-glucuronide

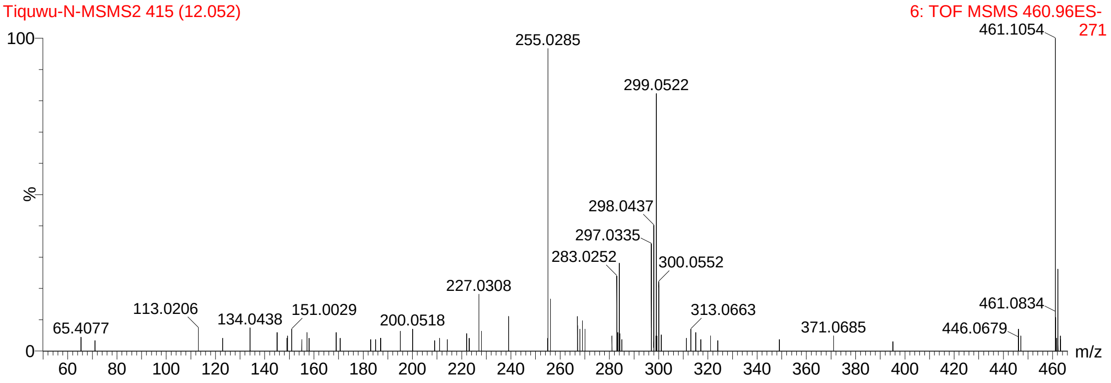

13 Chrysoeriol 7-O-glucoside

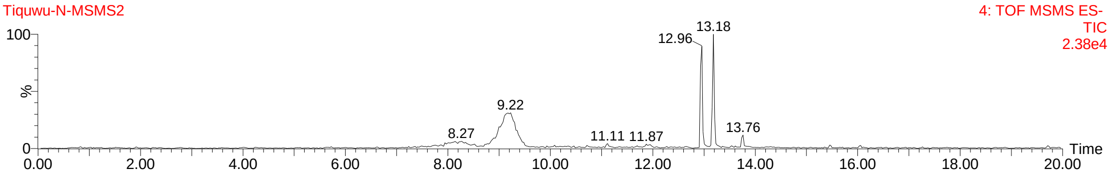

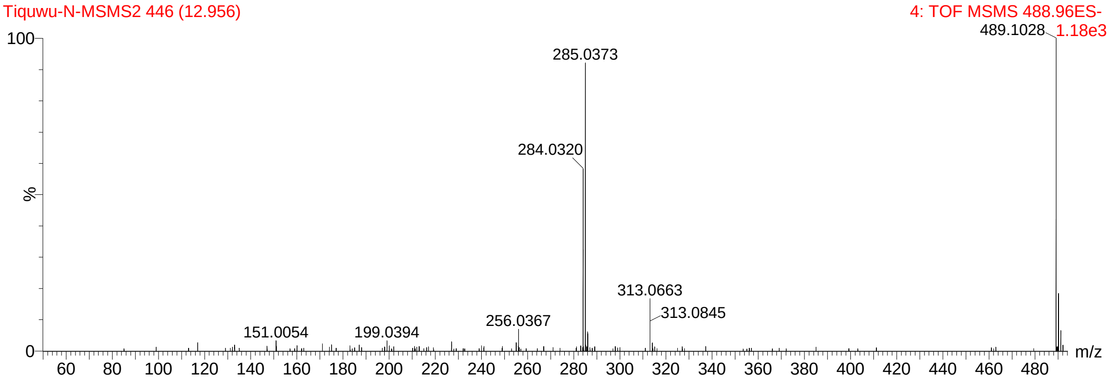

14 Luteolin 7-O-(6″-O-acetyl)-glucoside

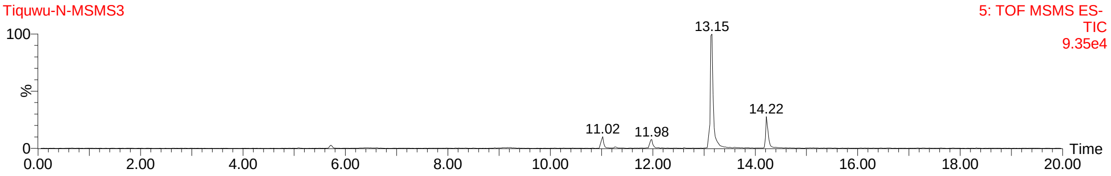

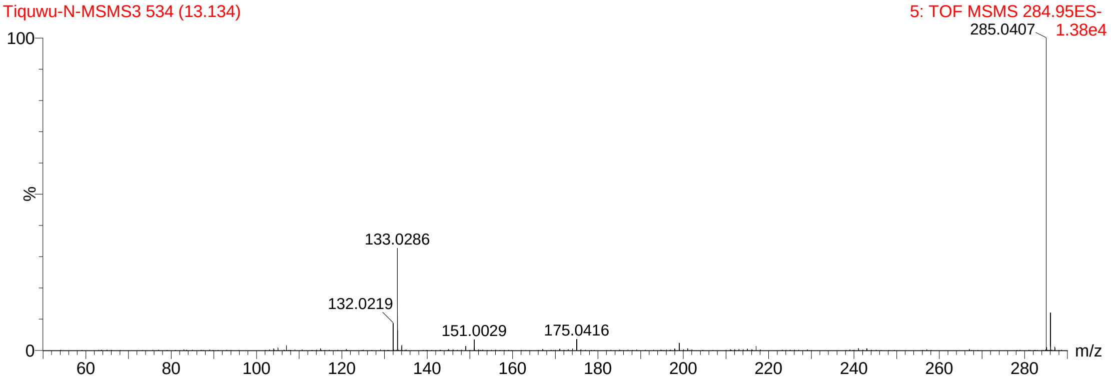

15 Luteolin

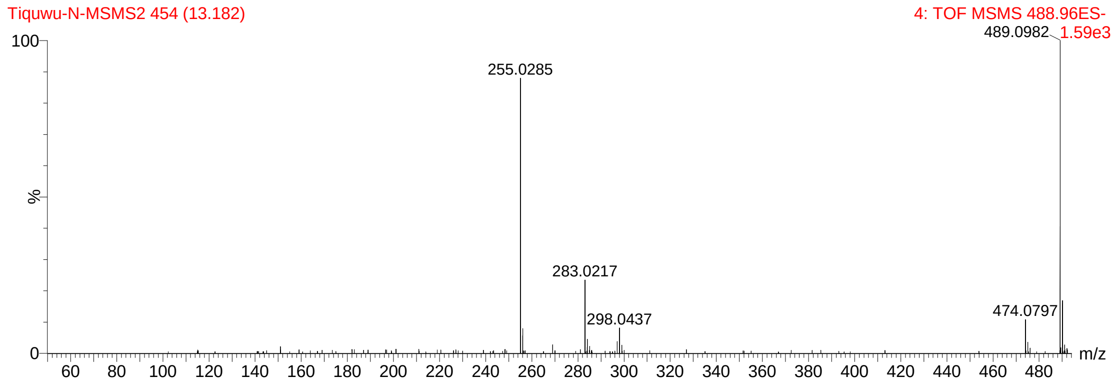

16 Chrysoeriol 7-O-methylglucuronide

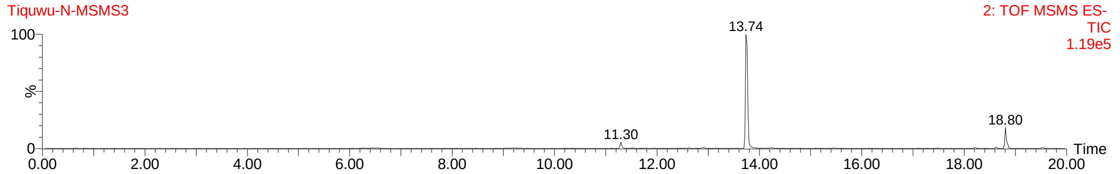

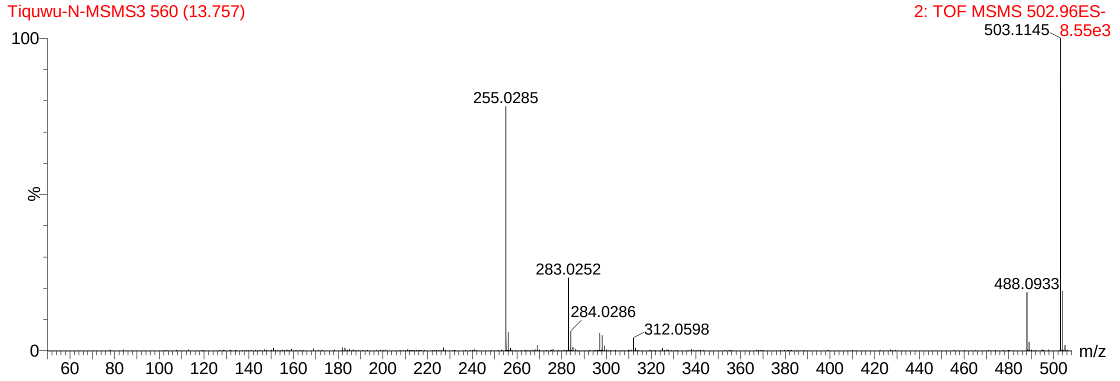

17 Chrysoeriol 7-O-(6″-O-acetyl)-glucoside

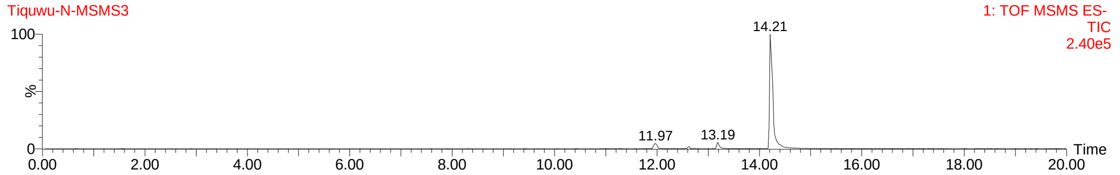

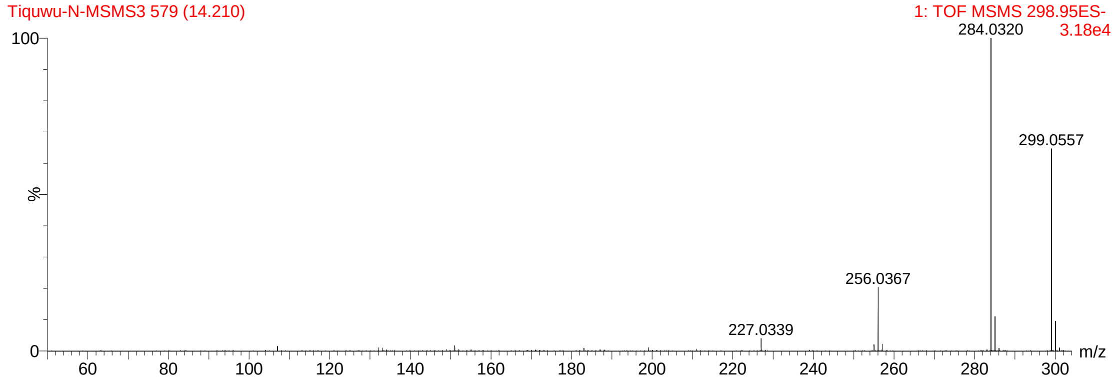

18 Chrysoeriol

$[M+H]^+$ (m/z)

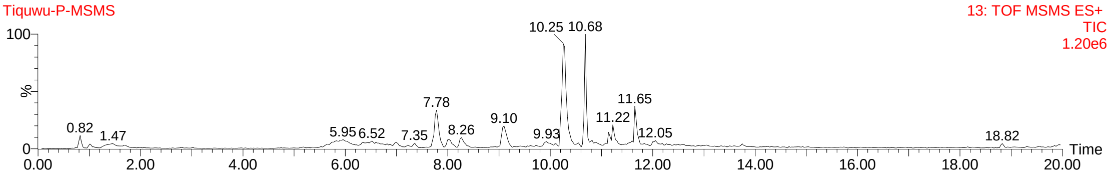

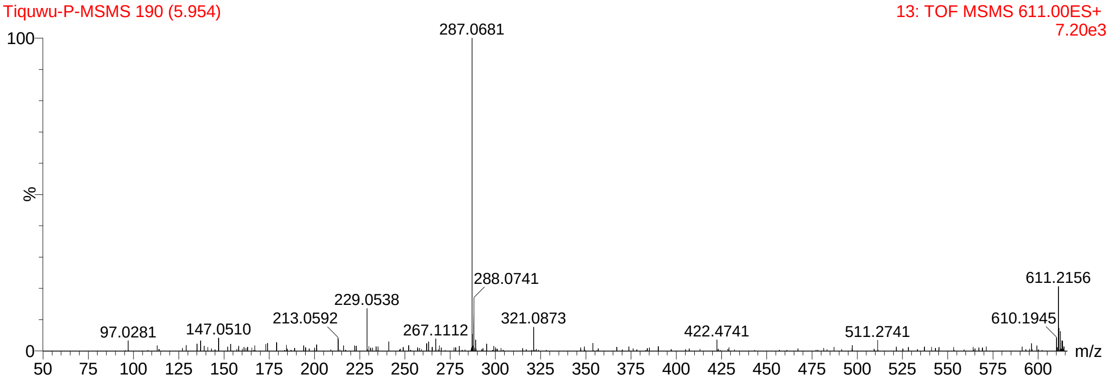

19 Cyanidin 3-O-diglucoside

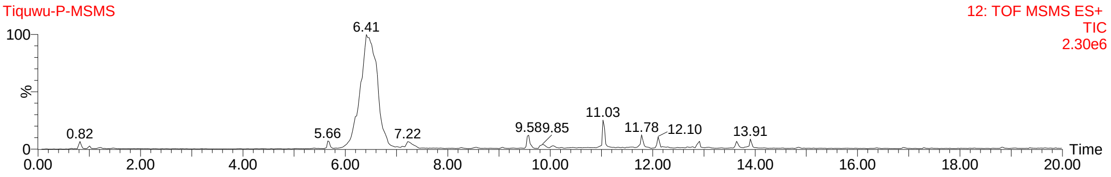

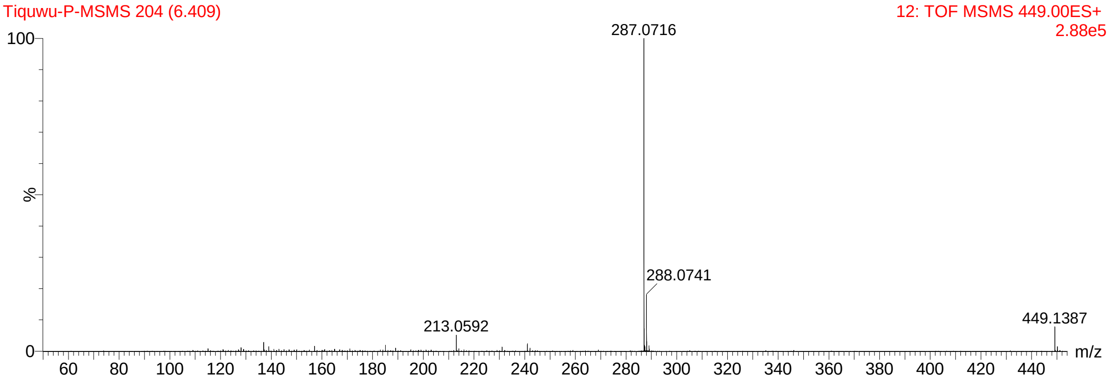

20 Cyanidin 3-O-glucoside

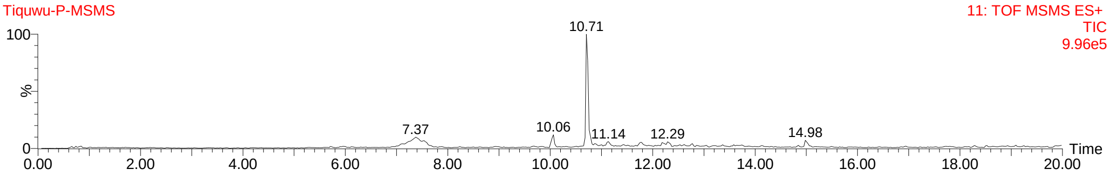

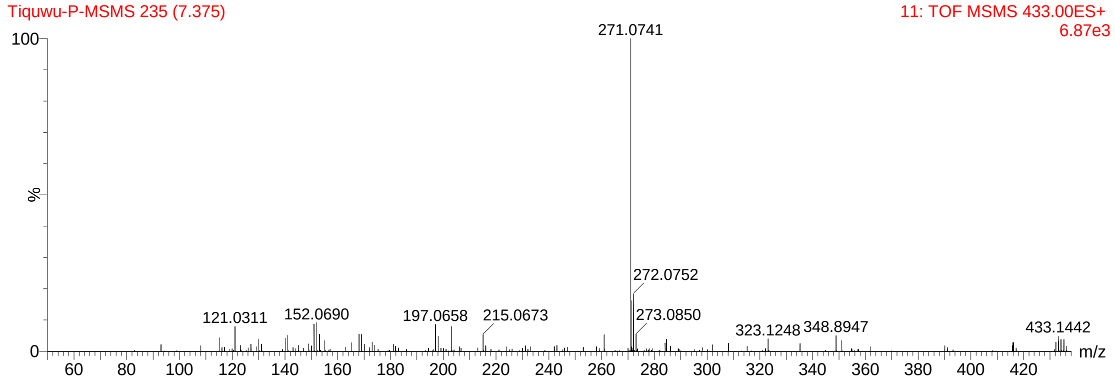

21 Pelargonidin 3-O-glucoside

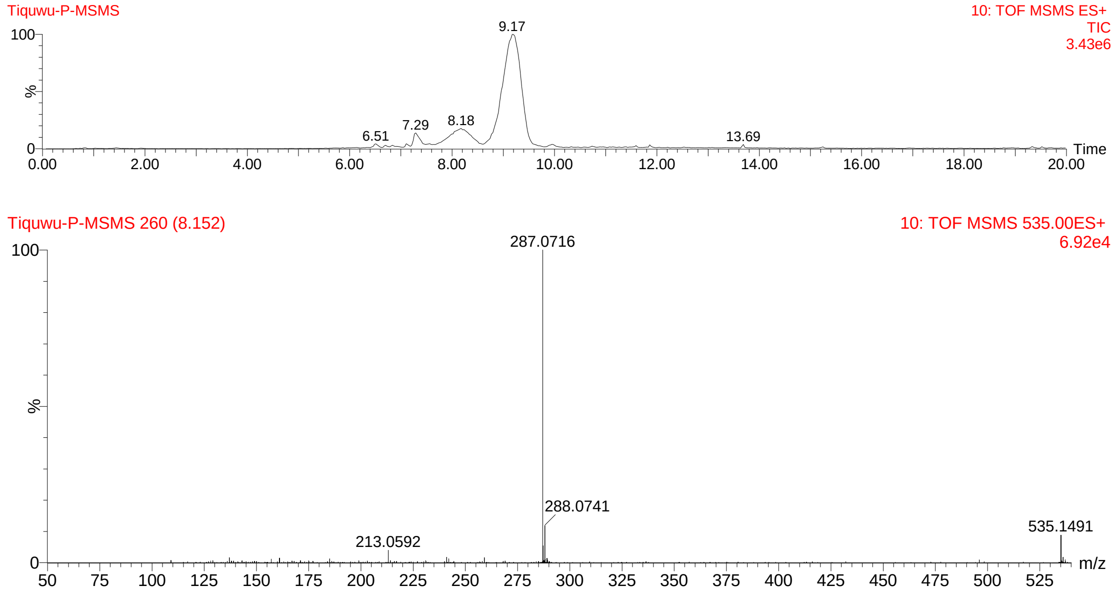

22 Cyanidin 3-O-(6″-O-malonyl)-galactoside

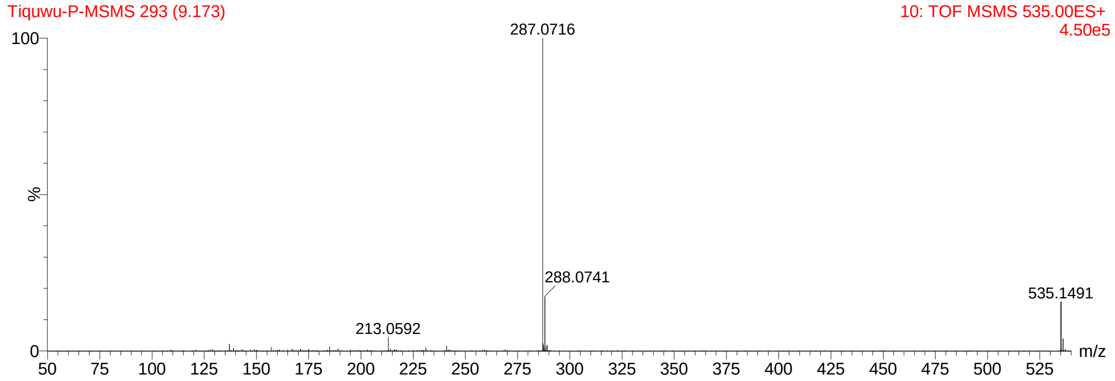

23 Cyanidin 3-O-(6″-O-malonyl)-glucoside

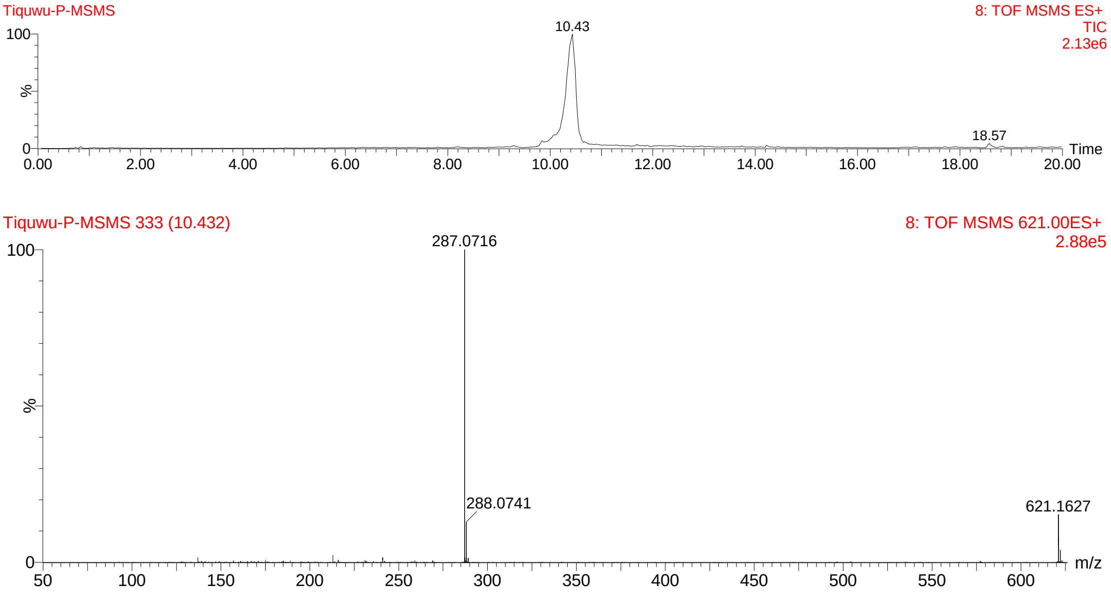

24 Cyanidin 3-O-(3″,6″-O-dimalonyl)-glucoside

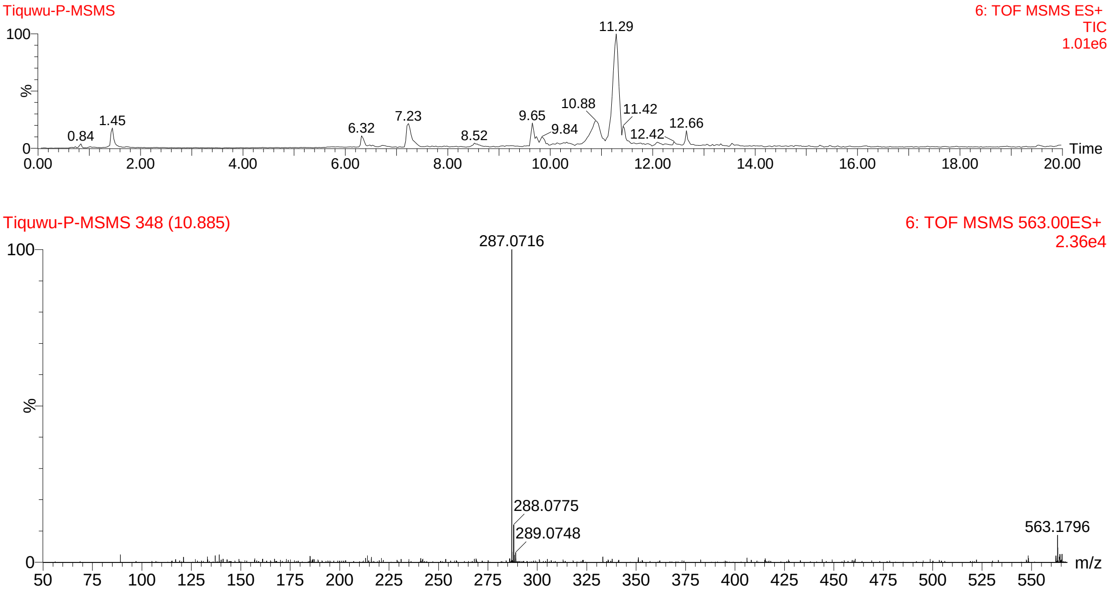

25 Cyanidin 3-O-(6″-O-succinyl)-glucuronide

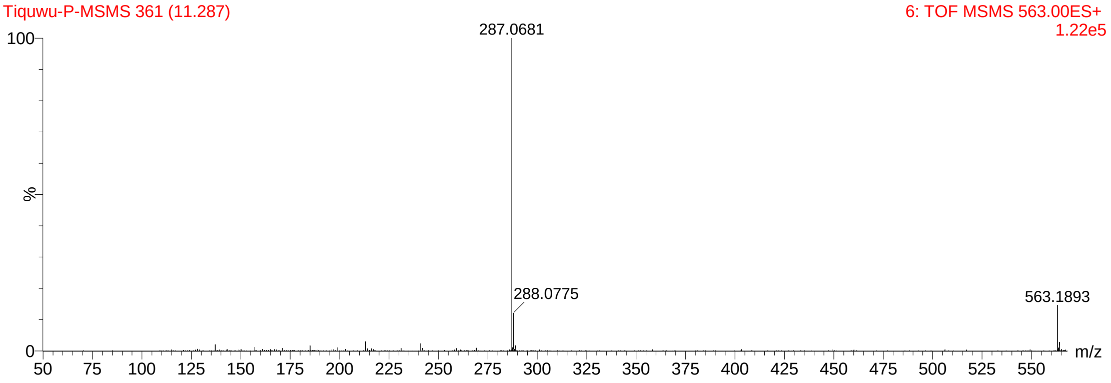

26 Cyanidin 3-O-(3″-O-succinyl)-glucuronide

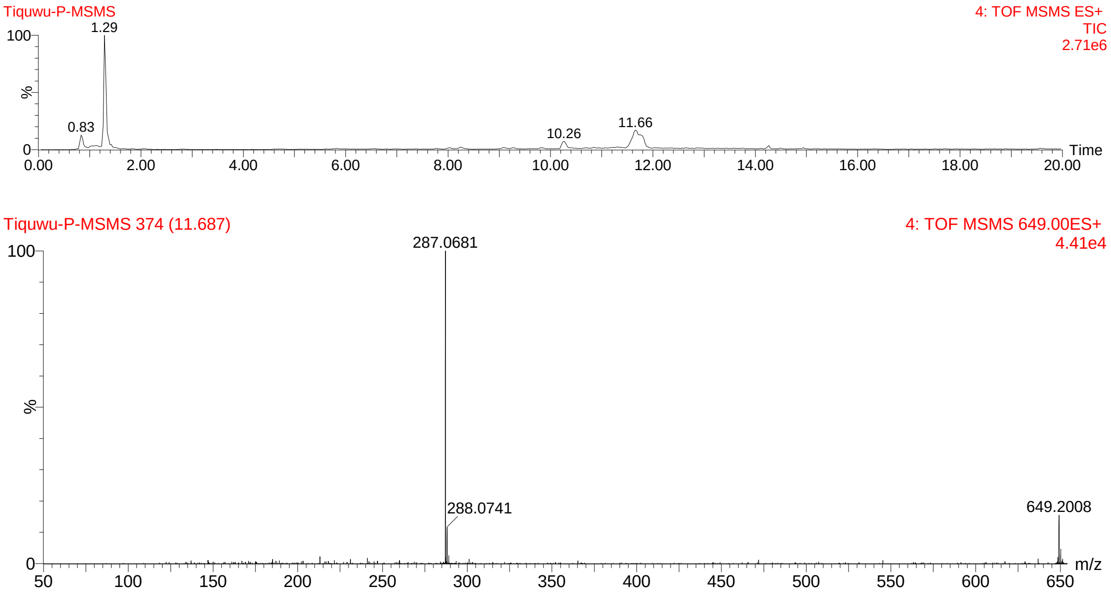

27 Cyanidin 3-O-(3″,6″-O-disuccinyl)-glucoside

Supplemantary Figure S2

The effects of HBP on the contents of SCFAs

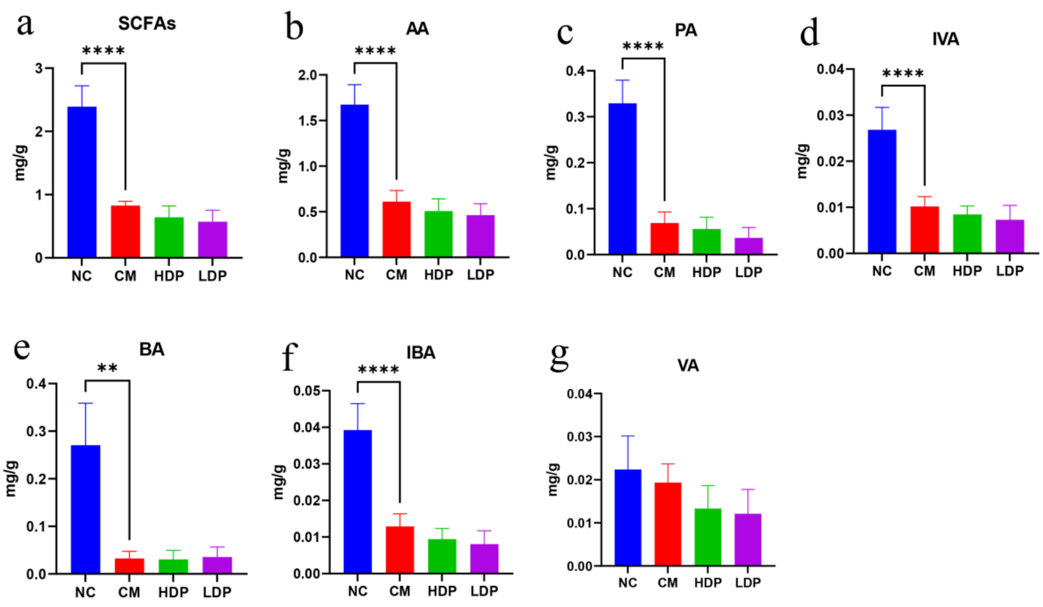

Supplementary Figure S3

KEGG functional enrichment analysis of HDF *vs* CM

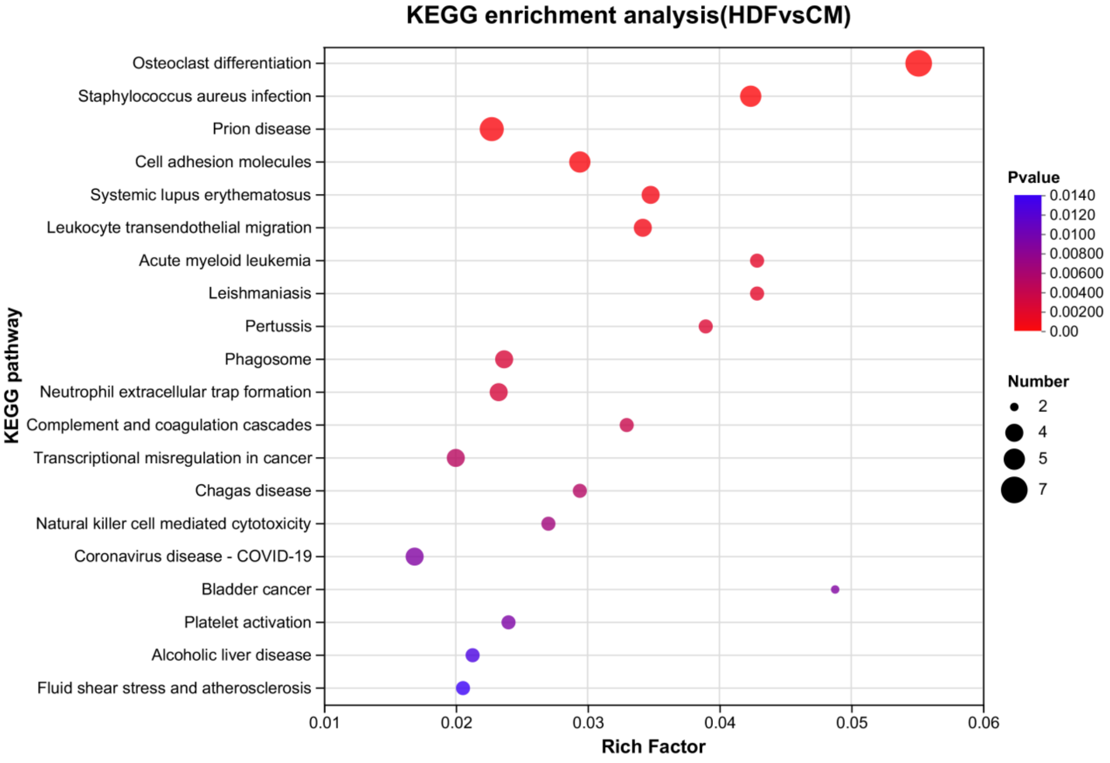

Supplementary Figure S4

IPP of Degs from immune system and signaling transduction

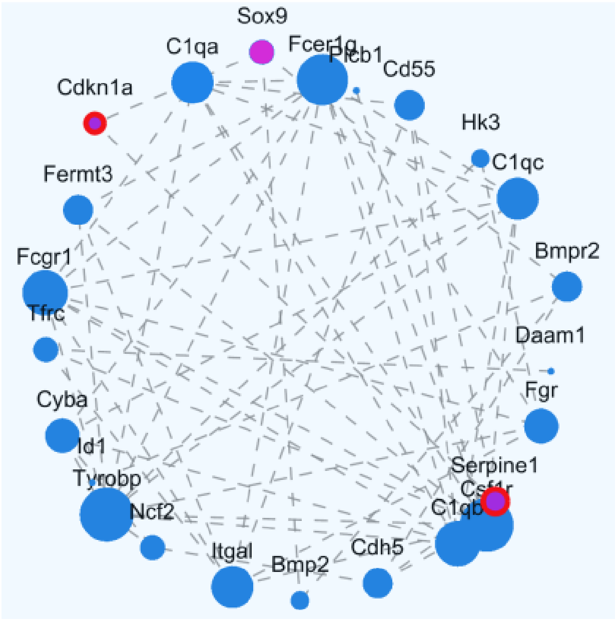

Supplement: Supplementary file 1 [file foods-14-02994-s001.zip › foods-3712774-supplementary.pdf]
